# Unmasking Methicillin-resistant *Staphylococcus argenteus*: pathogenic potential, diagnostic pitfalls and antibiotic resistance of MRSArg in Norway 2008–2019

**DOI:** 10.3389/fmicb.2025.1734191

**Published:** 2026-01-14

**Authors:** Torunn Gresdal Rønning, Hege Enger, Kyriakos Zaragkoulias, Christina Gabrielsen Ås

**Affiliations:** 1 Department of Medical Microbiology, Clinic of Laboratory Medicine, St. Olavs Hospital, Trondheim University Hospital, Trondheim, Norway; 2 The Norwegian MRSA Reference Laboratory, Department of Medical Microbiology, Clinic of Laboratory Medicine, St. Olavs Hospital, Trondheim University Hospital, Trondheim, Norway; 3 Department of Clinical and Molecular Medicine, Norwegian University of Science and Technology, Trondheim, Norway

**Keywords:** diagnostics, epidemiology, genomics, methicillin-resistance, MRSArg, screening, *Staphylococcus argenteus*, surveillance

## Abstract

**Background:**

*Staphylococcus argenteus* is a coagulase-positive species within the *Staphylococcus aureus* complex that may carry the *mecA* gene, conferring methicillin resistance. Despite its clinical relevance, methicillin-resistant *S. argenteus* (MRSArg) remains underreported and poorly characterized in many regions.

**Aim:**

This study presents the first nationwide analysis of MRSArg in Norway, detected among MRSA 18,152 isolates submitted to the Norwegian MRSA Reference Laboratory between 2008 and 2019.

**Methods:**

Clinical and epidemiological data were retrieved from national surveillance systems and laboratory records, enabling classification into healthcare- and community-associated categories. A representative panel of MRSArg genotypes (*n* = 25) was inoculated onto four MRSA-selective chromogenic agars for qualitative assessment of growth characteristics and colony pigmentation, and into two enrichment broths for quantitative evaluation of stationary-phase cell density. Identification was performed using MALDI-ToF MS and GeneXpert assays. Whole genome sequencing of 160 isolates enabled phylogenetic analysis, resistance and virulence profiling, and cgMLST. A species-specific multiplex PCR targeting *yhfT*, *mecA*, and *lukE*, along with adapted *spa*-typing primers, was developed for accurate molecular identification and typing.

**Results:**

A total of 160 *mecA*-positive MRSArg strains (0.9%) were identified, corresponding to 142 unique patients. Most cases were community-associated and linked to international acquisition, particularly from Southeast Asia. However, 36% were healthcare-associated, including a notable proportion among healthcare workers. The dominant genotype was *spa*-type t6675/ST2250 (50%), with increasing diversity observed after 2014. A species-specific multiplex-PCR and sanger sequencing primers for detection and genotyping of MRSArg were developed and validated.

**Conclusion:**

MRSArg is an emerging pathogen in Norway with both community and healthcare relevance. Improved molecular diagnostics are essential for accurate detection and surveillance.

## Introduction

*Staphylococcus argenteus* is a coagulase-positive species within the *Staphylococcus aureus* complex, formally described in 2015 as taxonomically distinct from *S. aureus* (Tong et al., 2015). The species was first identified in 2006 in Darwin, Northern Territory, Australia, following its isolation from the blood culture of a 55-year-old Indigenous Australian woman presenting with bacteraemia. Initially, the isolate was misclassified as an atypical *S. aureus* strain due to its close phenotypic resemblance, including colony morphology and biochemical characteristics. Subsequent molecular and phylogenetic analyses however, revealed significant genomic divergence, supporting its recognition as a separate species within the *S. aureus*-related clade (Tong et al., 2015). Still, *S. argenteus* shares a substantial portion of its genome with *S. aureus*, including in certain instances genes associated with antimicrobial resistance such as *mecA*, which confers resistance to methicillin and other β-lactam antibiotics.

Diagnostic procedures have until recently had limited ability to differentiate *S. argenteus* from *S. aureus* due to similarities in phenotypic characteristics, biochemistry and protein profiles. Furthermore, the pathogenic potential, epidemiology and clinical importance of *S. argenteus* is still unclear (McDonald et al., 2006; Okuma et al., 2002). Some studies have indicated that *S. argenteus* could be less virulent than *S. aureus*, due to the fact that *S. argenteus* lacks staphyloxanthin, a golden pigment recognized as an important virulence factor of *S. aureus* (Holt et al., 2011; Zhang et al., 2016). *S. argenteus* has furthermore been shown to carry fewer exotoxins and is usually susceptible to most antibiotics (Chantratita et al., 2016). Nevertheless, recent studies from Australia and Thailand have suggested that *S. argenteus* may cause human infections with a similar pathogenic potential as *S. aureus* (Chantratita et al., 2016; Thaipadungpanit et al., 2015).

Until recently, Matrix-Assisted Laser Desorption/Ionization Time-of-Flight Mass Spectrometry (MALDI-TOF MS), the most widely employed diagnostic method for microbial pathogen identification, was unable to distinguish between *S. argenteus* and *S. aureus*. This limitation stemmed from the absence of *S. argenteus* in reference databases of the commercial producers of MALDI-TOF MS systems, frequently resulting in misclassification of *mecA* positive *S. argenteus* (MRSArg) as methicillin-resistant *S. aureus* (MRSA) (Chen et al., 2018). In 2018, Bruker Daltonics addressed this issue by updating its MALDI Biotyper database to include both *S. argenteus* and *Staphylococcus schweitzeri* (RUO database 2018, V8.0, 7854 Bruker; Bruker Daltonics) (Eshaghi et al., 2021). However, not all commercially available MALDI-TOF MS platforms currently support this level of taxonomic resolution; for example, the VITEK MS system by bioMérieux lacks a database capable of distinguishing members of the *S. aureus* complex (Alhussein et al., 2025).

Since 2005 and 2019, respectively, laboratory-confirmed MRSA and MRSArg infections and colonizations have been notifiable in Norway. All isolates are confirmed and genotyped by *spa*-typing at the Norwegian MRSA Reference Laboratory at St. Olavs Hospital, as part of the national surveillance effort. This has facilitated the assessment of misclassified MRSArg cases among an extensive collection of MRSA isolates. In this study, we aimed to quantify the extent of misclassification and to characterize the clinical, molecular, and epidemiological features of methicillin-resistant *Staphylococcus argenteus* in Norway. Furthermore, we sought to assess and develop diagnostic tools for its rapid identification and genotyping. By combining genomic and epidemiological characterization with the development of novel molecular methods, this work expands scientific knowledge of *S. argenteus*, supports accurate species-level identification, and enhances diagnostic precision. This work contributes to national surveillance and may inform infection control strategies within clinical microbiology.

## Results

### Epidemiology and trends of MRSArg

**FIGURE 1:**
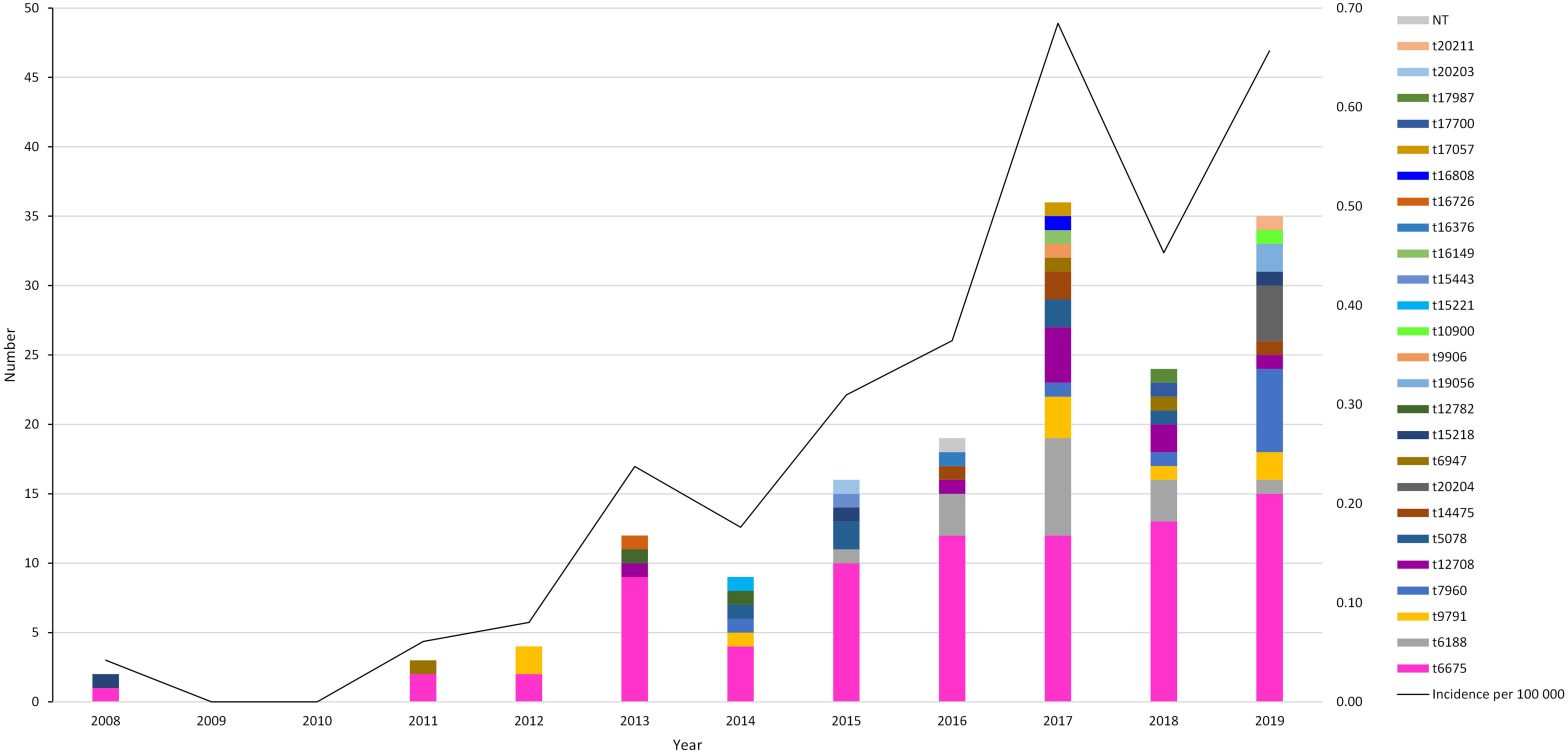
Yearly number of MRSArg strains (*y*-axis) detected during the study period (2008–2019), with *spa*-type indicated by the colored key. Incidence per 100,000 population is indicated by the black line, corresponding to the right axis. NT: Non-typable.

From a total of 18,152 MRSA isolates collected between 2008 and 2019, 160 *mecA*-positive MRSArg strains were identified (0.9%), corresponding to 142 unique patients. This corresponds to a relatively low yearly incidence of 0.0–0.7 per 100,000 population (Figure 1).

The earliest detection of MRSArg isolates occurred in 2008, corresponding to the initial year of the study period. An increasing trend in the prevalence of MRSArg was observed over the course of the study period (Figure 1), rising from only two isolates in 2008 to 35 isolates in 2019. The most frequently detected *spa*-type across the whole study period was t6675 (*n* = 80, 50%), and the diversity of *spa*-types among MRSArg isolates expanded after 2014.

The age of MRSArg cases ranged from 0 to 89 years, with a median age of 33 years (Table 1). The sex distribution was significantly skewed toward female patients (59%) compared to the sex distribution among MRSA in the same period.

**TABLE 1:** Clinical and epidemiological information on all MRSArg cases in Norway in 2008–2019.

Clinical and epidemiological categories			This study (2008–2019)		Rønning et al. (2008–2017) (Ronning et al., 2024)		Significance (*p*-value)
			n	%	n	%	
Samples	Total samples		160	100	15,200	100	
	Total patients		142	100	14,386	100	
	Female		94	59	7,173	50	0.03
	Male		66	41	7,211	50	
Carriage/infection	Carriage		121	76	8,162	54	<0.001
	Infection		38	24	5,498	36	
	NA		1	0.6	1,540	10	
HA/CA	Healthcare associated	Total	58	36	4,566	30	
		Admitted to hospital	27	17	3,004	20	
		Health care worker	27	17	933	6	<0.001
		Other	4	3	629	4	
	Community associated	Total	102	64	10,634	70	
Place of acquisition	Europe	Total	53	33	5,095	34	
		Norway	49	31	4,199	28	
	Asia	Total	68	43	1,872	12	
		Philippines	57	36	187	10	<0.001
		Vietnam	8	5	NA	NA	
	Africa	Total	5	3	430	3	
	America	Total	2	1	302	2	
	Unknown	Total	32	20	7,123	47	

Number of cases and percentages are given with values rounded to the nearest whole number. Association to country is included for countries with cases where *n* ≥ 5. Significant differences between the studies have *p*-values written in the table.

The majority of MRSArg detected in this study were related to carriage (76%). This was significantly higher than previously reported for MRSA isolates during the same study period (Table 1). Infections constituted 24%, and no invasive infections were reported. Community-associated cases constituted the majority (64%) of cases, while 36% of MRSAarg cases exhibited an epidemiological connection to the healthcare system at the time of sampling, qualifying them as HA-MRSArg. Notably, 17% of individuals were identified as health care workers (HCWs), which was significantly higher compared to MRSA cases during the same study period. However, only two strains (1.3%) were reported as outbreak associated.

Analysis of acquisition origin revealed that Asia constituted the predominant region, accounting for 43% of cases. Within Asia, the Philippines represented the most significant country-specific source, contributing 36% of total acquisitions. Europe, excluding Norway, comprised 2.5% of cases. Acquisition within Norway was reported for 31% of instances. Overall, the country of acquisition was registered in 80% of cases.

### Species identification

Although the Bruker Daltonics MALDI-TOF database was updated to include *S. argenteus* in 2018, our results show that MALDI-TOF MS exhibited limited discriminatory power in differentiating *S. argenteus* from especially the most closely related staphylococci like *S. aureus* and *S. schweitzeri* (Table 2). Of the tested representative collection of strains (*n* = 25), three strains were identified as *S. aureus* (11.1%), two as *S. schweitzeri* (7.4%) and one strain did not get any species ID (3.7%). Two additional strains were identified as *S. argenteus*, but with scores below 2.0 (7.41%). Thus, 29.6% of strains were either not correctly identified or had too low score for species-level identification, while 70.4% of strains were identified correctly to species level.

**TABLE 2:** Results from growth on commercially available chromogenic media, rapid detection of MRSA using the Xpert SA Nasal Complete kit (Cepheid) and MALDI-TOF MS (Bruker Daltonics) of MRSArg strains.

*spa*-type	MLST	GeneXpert MRSA	MALDI-TOF MS best hit including score	MALDI-TOF MS second best hit including score	chromID MRSA SMART	Scientific brilliance MRSA2	BD BBL CHROMagar MRSA II	CHROMagar TM MRSA	TSB	TSBM	PHMB
t15221	ST1223	NT	*S. argenteus*, score 2.09	*S. schweitzeri*, score 2.09	+	+	+	+	NT	NT	NT
t15218	ST1223	NT	*S. argenteus*, score 1.96	*S. argenteus*, score 1.93	+	+	+	+	NT	NT	NT
t9906	ST1223	MRSA positive	No sure ID, score 1.50	No sure ID, score 1.47	+	+	+	+	NT	NT	NT
t15443	ST1223	NT	*S. argenteus*, score 2.44	*S. argenteus*, score 2.22	+	+	+	+	NT	NT	NT
t12783	ST1223	NT	*S. schweitzeri*, score 1.83	*S. argenteus*, score 1.78	+	+	+	+	NT	NT	NT
t9791	ST1223	MRSA positive	*S. argenteus*, score 2.12	*S. argenteus*, score 2.06	+	+	+	+	+	+	+
t16726	ST2793	NT	*S. argenteus*, score 2.10	*S. argenteus*, score 2.06	+	+	+	+	NT	NT	NT
t17700	ST2793	NT	*S. aureus*, score 2.0	*S. argenteus*, score 1.95	+	+	+	+	NT	NT	NT
t6188	ST2793	MRSA positive	*S. aureus*, score 1.74	No sure ID, score 1.69	+	+	+	+	+	+	+
t19056	ST2793	NT	*S. argenteus*, score 2.16	*S. argenteus*, score 2.13	+	+	+	+	NT	NT	NT
t17987	ST2793	NT	*S. argenteus*, score 2.0	*S. argenteus*, score 1.95	+	+	+	+	NT	NT	NT
t6947	ST2793	MRSA positive	*S. argenteus*, score 2.18	*S. argenteus*, score 2.14	+	+	+	+	NT	NT	NT
t17057	ST2793	NT	*S. argenteus*, score 2.18	*S. argenteus*, score 2.16	+	+	+	+	NT	NT	NT
t20203	ST2250	NT	*S. aureus*, score 2.37	*S. argenteus*, score 2.37	+	+	+	+	NT	NT	NT
t20211	ST2250	NT	*S. argenteus*, score 1.97	*S. argenteus*, score 1.86	+	+	+	+	NT	NT	NT
t20204	ST2250	NT	*S. argenteus*, score 2.0	*S. argenteus*, score 1.95	+	+	+	+	NT	NT	NT
t14475	ST2250	NT	*S. argenteus*, score 2.11	*S. argenteus*, score 2.06	+	+	+	+	NT	NT	NT
t16376	ST2250	NT	*S. argenteus*, score 2.21	*S. argenteus*, score 2.20	+	+	+	+	NT	NT	NT
t12708	ST2250	NT	*S. argenteus*, score 2.20	*S. argenteus*, score 2.13	+	+	+	+	NT	NT	NT
t6675	ST2250	MRSA positive	*S. schweitzeri*, score 1.82	*S. argenteus*, score 1.78	+	+	+	+	+	+	+
t7960	ST2250	NT	*S. argenteus*, score 2.18	*S. argenteus*, score 2.14	+	+	+	+	NT	NT	NT
t16149	ST2250	NT	*S. argenteus*, score 2.21	*S. argenteus*, score 2.16	+	+	+	+	NT	NT	NT
t16808	ST2250	NT	*S. argenteus*, score 2.12	*S. argenteus*, score 2.11	+	+	+	+	NT	NT	NT
t5078	ST2250	MRSA positive	*S. argenteus*, score 2.13	*S. argenteus*, score 2.12	+	+	+	+	+	+	+
t10900	ST2250	NT	*S. argenteus*, score 2.11	*S. argenteus*, score 2.09	+	+	+	+	NT	NT	NT

NT, not tested; TSB, Tryptic Soy Broth; TSBM, Tryptic Soy Broth Modified with aztreonam and cefoxitin; PHMB, Phenolred Mannitol Broth with ceftizoxime and aztreonam. “+” marks growth.

*In silico* species identification with Kraken2 in combination with the PlusPF database also exhibited limited accuracy in correctly identifying *S. argenteus* (Table 3). For only 25.5% of strains, *S. argenteus* was the top species match, with a median percent identity of only 50.7%. These included ST2793 and ST1223. For 74.5% of strains however, *S. aureus* was the best match, with a very low median identity of 4.6%, and these strains were ST2250.

**TABLE 3:** Kraken2 taxonomic classification of the MRSArg-strains analyzed in this study.

Species and ranked match	Strains (n)	Strains (%)	Median identity (%)	STs
*S. argenteus* #1 match	40	25.5	50.7	2,793, 1,223
*S. aureus* #2 match	39	24.8	6.7	2,793, 1,223
*S. aureus* #1 match	117	74.5	4.6	2,250
*S. argenteus* #2 match	110	70.1	2.6	2,250
Unclassified #1 match	0	0.0	0.0	
Unclassified #2 match	8	5.1	2.7	2,250, 1,223

### Screening and enrichment

For the panel of MRSArg strains representing all *spa*-types in this study (*n* = 25), growth was observed on all the chromogenic agar media tested in this study (Supplementary Figure 1). With inoculation of a standardized concentration of MRSArg, chromID MRSA SMART agar yielded the highest growth, or equivalent growth to another chromogenic medium, in 21 out of 25 tested strains (84%). These findings indicate that chromID MRSA SMART agar was thus the most sensitive agar among those assessed for detection of MRSArg.

**FIGURE 2:**
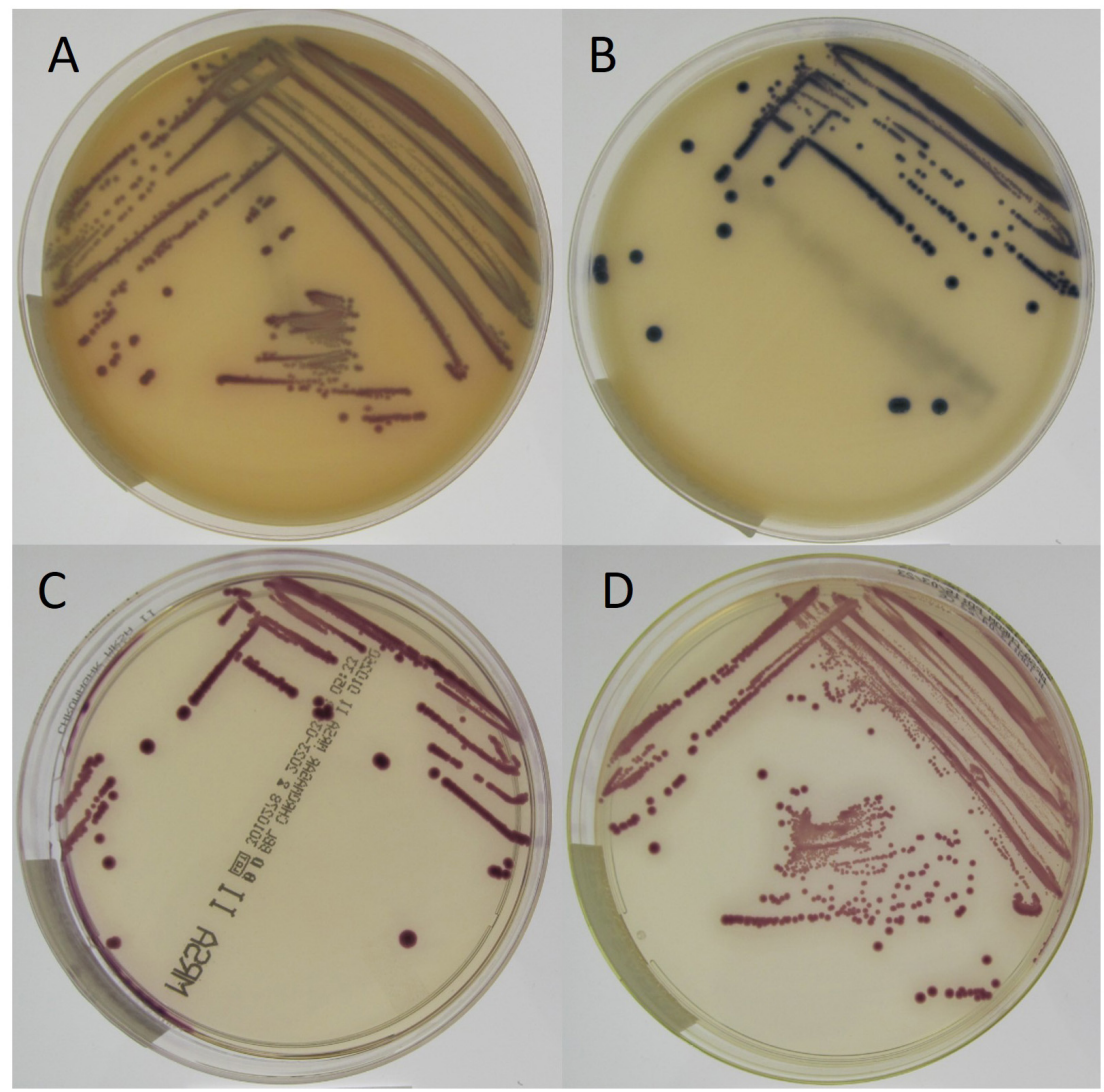
Growth of MRSArg t6675/ST2250 strain SO-SARG15-1 on all evaluated chromogenic agars after 48 h of incubation. **(A)** chromID MRSA SMART agar, **(B)** Scientific Brilliance MRSA2 agar, **(C)** BD BBL Chromagar MRSA II agar, and **(D)** CHROMagar TM MRSA.

On all media except chromID MRSA SMART, the colony coloration of MRSArg closely resembled that of MRSA (Figure 2; Supplementary Figure 1). On chromID MRSA SMART agar however, MRSArg colonies exhibited a blueish pigmentation following 48 h of incubation, in contrast to the characteristic pink coloration of MRSA colonies on the same agar. These findings suggest that chromID MRSA SMART agar may serve as a useful supplementary tool for phenotypic differentiation between MRSA and MRSArg.

Results from evaluation of the two enrichment broths for growth of MRSArg showed that growth in PHMB yielded the highest average stationary-phase cell density, reaching 2.67 × 10^8^ CFU/mL (Supplementary Table 1). These results furthermore highlight considerable variation in stationary-phase concentrations across the evaluated broths, with PHMB demonstrating notably higher yield (170%) relative to TSB, while growth in TSBM yielded very low (2%) stationary-phase cell density relative to TSB.

#### Rapid identification using GeneXpert

All selected MRSArg strains (*n* = 7), representing the three distinct sequence types previously identified in this study, yielded positive results for identification of MRSA using the rapid identification assay Xpert SA Nasal Complete on the GeneXpert XVI instrument (Table 2). The strains were positive for all targets, including *spa*, *mecA*, and SCC*mec*. These results indicate that although Xpert SA Nasal Complete does not distinguish between MRSA and MRSArg, it could be used for rapid detection of MRSArg.

#### Evaluation of multiplex PCR for confirmation of MRSArg

Evaluation of the multiplex real-time PCR assay targeting the *yhfT*, *lu*k*E*, and *mecA* genes demonstrated complete concordance with WGS results across all tested MRSArg genotypes (*n* = 20) (Supplementary Table 2). Of note, 5/20 MRSArg isolates were negative for *lukE* by multiplex PCR while remaining positive for *yhfT*, confirming that *yhfT* represents the primary marker for MRSArg detection in this assay and that *lukE* should be regarded as a secondary, non-universal target among MRSArg genotypes. All tested MRSA genotypes (*n* = 20) yielded negative results for both *yhfT* and *lukE* targets, thereby supporting the specificity of these markers. Further specificity testing against other species was not performed. Because the primers and probe for *lukE* were designed specifically to detect *lukE* in *S. argenteus*, they had multiple mismatches against the *lukE* gene of the *S. aureus* Newman reference strain, encoding *S. aureus* LukED. All target genes furthermore demonstrated high amplification efficiency, with values of 95.5% for *yhfT*, 95.7% for *lukE* and 95.7% for *mecA* (Table 4). The sensitivity of the combined PCR for all three targets was determined to be 47.1 copies per PCR reaction. Both extraction methods produced comparable multiplex-PCR amplification results under identical conditions. These findings indicate that either preparation method can be reliably applied for the multiplex PCR. Collectively, these results indicate that the developed multiplex PCR assay represents a promising tool for the detection of MRSArg and for its differentiation from MRSA.

**TABLE 4:** Performance metrics of multiplex real-time PCR targeting the *yhfT*, *lukE*, and *mecA* genes.

Strain	Gene	Efficiency (%)	Sensitivity (copies/PCR)
*S. argenteus* t6675/ST2250 SO-SARG8-1	*yhfT*	95.53	47.1
	*lukE*	95.68	47.1
	*mecA*	95.71	47.1

#### *spa*-typing of MRSArg

**FIGURE 3:**
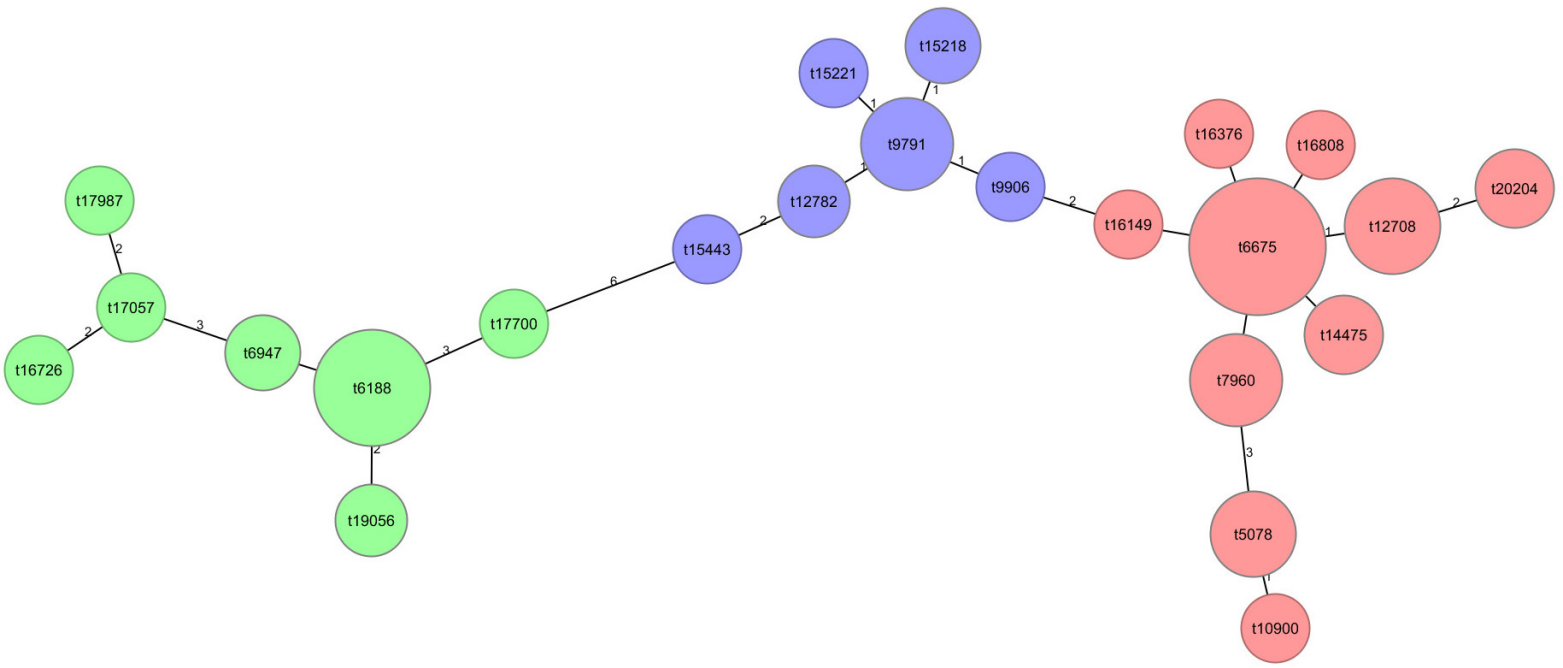
Minimal spanning tree of the MRSArg *spa*-types included in this study. The MST illustrates genetic relationships among *spa*-types based on the *spa*-repeat similarity. Each node represents a unique *spa*-type, and edges indicate the number of repeat differences. Node size is proportional to the number of isolates represented, with larger nodes indicating a higher isolate count. The nodes are color-coded into three major clusters corresponding to different sequence types (STs): green for ST2793, blue for ST1223, and red for ST2250.

Novel *spa*-typing primers for *S. argenteus* were developed to address sequence mismatches with existing *S. aureus*-based primers, which consistently failed to yield successful *spa*-typing results for MRSArg strains. Implementation of the newly optimized primers (Table 5) enabled successful Sanger sequencing-based *spa*-typing across the entire strain collection, achieving 100% typability. A minimal spanning tree (MST) based on *spa*-repeat sequences of all the Sanger-sequenced strains illustrates the relatively low *spa*-type variability of MRSArg strains in this study, with three main clusters representing the three different sequence types, ST2250 (*n* = 117), ST1223 (*n* = 16) and ST2793 (*n* = 24) (Figure 3).

**TABLE 5:** Primer and probe sequences for MRSArg-multiplex PCR, and primer pairs targeting the X-region of the *spa* gene in *Staphylococcus argenteus*.

Target gene	Forward primer (5’–3’)	Reverse primer (5’–3’)	Probe (5’–3’)	Position (5’–3’) in ref. strain ASM2315891v1	References
*mecA* (Nakagawa et al., 2005)	*mecA*-f: TGGTATGTGGAAGTTAGATTGGGAT	*mecA*-r: CTAATCTCATATGTGTTCCTGTATTGGC	*mecA*-probe: JUN-TCCAGGAATGCAGAAAG ACCAAAGCA-QSY	f: 43,672–43,696 r: 43,542–43,569 p: 43,632–43,658	(Pantosi and Venditti, 2009)
*yhfT*	*yhfT*-f: GACAACCGGCATGCCAAAAG	*yhfT*-r: AGAATGTGCAAGTGGTCCAG	*yhfT*-probe: VIC-AGCGTGGAGAAAATGCC ATTGTAGC-MGBNFQ	f: 607,550–607,569 r: 607,657–607,676 p: 607,630–607,654	This study
*lukE*	*lukE*-f: CGGTCAAAAGTCAGCACATG	*lukE*-r: AGGTGGCAGTTGGTTATCAG	*lukE*-probe: 6FAM-CCCAACAGGTTCAGCA CGAGAGTA-MGBNFQ	f: 1,896,258–1,896,277 r: 1,896,175–1,896,194 p: 1,896,203–1,896,226	This study
*spa* (sanger sequencing)	*spa*-1113arg-f: TAAAGATGACCCAAGTCAAAGC	*spa*-1514arg-r: CAGCAGTTGTTCCATGTGCTT		r: 89,381– 89,402 f: 88,636–88,656	This study

#### Phenotypic antibiotic susceptibility and resistome

All strains (*n* = 160) were PCR positive for the *mecA* gene, which was encoded on SCC*mec* type IV. In addition, β-lactamase genes were detected in a majority of whole genome sequenced strains, including *blaZ* (*n* = 141, 89.8%) and *blaPC1* (*n* = 10, 6.3%) (Table 6). Consistent with the universal presence of *mecA*, phenotypic susceptibility testing showed resistance to cefoxitin in 100% of strains.

**TABLE 6:** Phenotypic susceptibility profiles of MRSArg-isolates in this study and resistance genes/mutations detected using AMRFinder plus.

Antibiotic	Total strains	Resistant strains (n)	Resistant strains (%)	Antibiotic resistance genes	Mutations conferring resistance	Comment
Cefoxitin	160	160	100.0%	*mecA, blaZ, blaPC1*	None detected	
Mupirocin	160	11	6.9%	None detected	None detected	All resistant strains close to breakpoint
Tetracycline	160	5	3.1%	*tet(K)*	None detected	
Clindamycin	160	4	2.5%	*erm(C)*	None detected	
Erythromycin	160	3	1.9%	*erm(C), msr(A)*	None detected	
Gentamicin	160	0	0.0%	None detected	None detected	
Rifampicin	160	1	0.0%	None detected	None detected	
Linezolid	160	0	0.0%	None detected	None detected	
Ciprofloxacin/norfloxacin	160	0	0.0%	None detected	None detected	
Fusidic acid	160	0	0.0%	None detected	None detected	
Trimethoprim-sulfametoxazol	160	0	0.0%	*dfrG*	None detected	Resistance gene only to the trimethoprim-component detected.
Vancomycin	160	0	0.0%	None detected	None detected	

Resistance to other antimicrobials was however, infrequent in the MRSArg strains (Table 6). Phenotypic resistance to mupirocin was detected in 6.9% of strains (*n* = 11), despite absence of known resistance genes or mutations. These resistant isolates however, exhibited inhibition zones close to the clinical breakpoint. Phenotypic resistance to tetracycline was identified in 3.1% (*n* = 5) of the tested isolates and was associated with the presence of *tet(K)* (*n* = 5). Resistance to clindamycin and erythromycin was observed at low levels, 2.5% (*n* = 4) and 1.9% (*n* = 3), respectively. The *erm(C)* gene was found in strains resistant to both antibiotics, while *msr(A)* was additionally detected in erythromycin-resistant isolates.

None of the isolates displayed resistance to gentamicin, rifampicin, linezolid, ciprofloxacin/norfloxacin, fusidic acid, or vancomycin (Table 6). For trimethoprim-sulfamethoxazole, no phenotypic resistance was observed. The *dfrG* gene, which confers resistance to the trimethoprim component, was detected in 133 (84.7%) of MRSArg strains, mainly in ST2250 (*n* = 110, 94.0%) and ST2793 (*n* = 23, 95.8%). ST1223 was significantly underrepresented (*n* = 0) among *dfrG*-positive isolates, indicating a strong negative association between this lineage and the presence of the gene (*p* < 0.01).

#### Core genome phylogeny and genomic diversity

**FIGURE 4:**
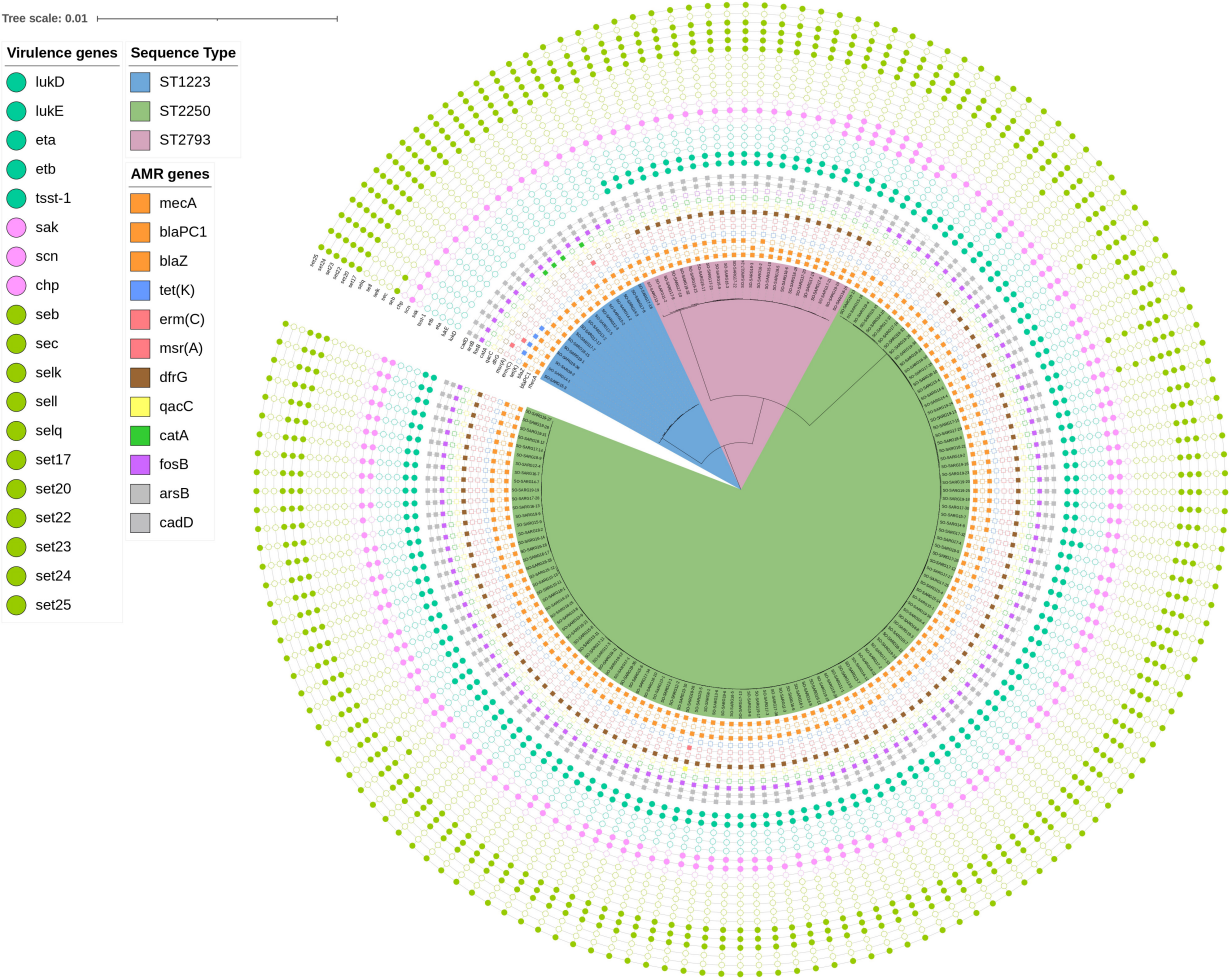
Core genome tree displaying the sequenced MRSArg in this study. Three distinct clades are shown colored according to their sequence types.

Whole genome sequencing emphasized the limited genomic diversity of MRSarg strains across the study collection. Core genome analysis (1,7 Mbp) yielded three distinct clades which corresponded to the three sequence types, ST2250, ST2793 and ST1223 (Figure 4). Core genome SNP analysis was furthermore performed to assess the genetic diversity within STs (Supplementary Table 3). Pairwise SNP distances were calculated for each ST, excluding closely related clusters (defined as ≤ 25 SNPs), to focus on broader population-level variation. ST2250 (*n* = 117) was the most prevalent lineage. Within this group, SNP distances ranged from 26 to 157, with a mean of 61 and an average of 65 SNPs. ST1223 (*n* = 16), exhibited the highest mean SNP distance (99) and average (96), suggesting greater intra-lineage heterogeneity. ST2793 (*n* = 24), showed the widest SNP range (35–245) and a mean of 79. No major related clusters (≤25 SNPs) (Raven et al., 2019) were detected, suggesting the absence of cryptic transmission events or outbreak-related strain groupings.

#### Virulence factors

MRSArg strains appeared to share many of the typical *S. aureus* virulence factors (Figure 4; Supplementary Table 4), with some variation between the different sequence types. In contrast, a subset of isolates, particularly those belonging to ST2793, showed a significant absence of the type-specific genes (Δ*cap8HIJK*, *p* < 0.01). A majority of strains furthermore encoded the polysaccharide intercellular adhesin (PIA) operon (*icaADBC*) involved in biofilm formation.

All MRSArg strains encoded a repertoire of extracellular enzymes, including lipase (*lip*), proteases (*aur*, *sspA*, *sspB*) and hyaluronate lyase (*hysA*). Near all ( > 99%) strains furthermore encoded multiple iron acquisition factors (*isdC*, *isdD*, *isdE*, *isdG*, *isdI*, *srtB*).

Of adhesion factors, most strains encode serine-aspartate (SD) repeat proteins *sdrC*, *sdrD* and *sdrE*, while clumping factor B (*clfB*) and collagen adhesin (*cna*) significantly associated with ST2793 (*p* < 0.01) and fibronectin-binding protein B (*fnbB*) mainly detected in ST2250 and ST2793.

All MRSArg strains encoded the ESAT-6-like secretion system (ESS), however, ST2250 strains were missing key structural (Δ*esxBCD*) and effector genes (Δ*esaCDE*).

Nearly all strains (>99%) carried the immune evasion-related genes adenosine synthase A (*asdA*) and the immunoglobulin-binding proteins *sbi* and *spa*. In contrast, the distribution of genes belonging to the immune evasion cluster (IEC; *sak, chp, scn*) showed considerable variability across MRSArg strains and sequence types (Figure 4; Supplementary Table 4). Notably, the *sak* gene was significantly underrepresented in ST1223 isolates, with no strains harboring this gene (*n* = 0, *p* < 0.01).

All MRSArg strains encoded alpha-, beta-, and delta-hemolysin genes (*hla*, *hlb*, *hld*) and near all (>99%) the gamma-hemolysin toxin (*hlgABC*). A majority of the MRSArg strains (91%) furthermore encoded a conserved leukocidin (*lukDE*) most similar to LukDE in *S. aureus* strain Newman, displaying protein level homology of 87–88 and 92.6% to LukD and LukE accordingly. A small number of strains (*n* = 16) additionally appeared to encode highly truncated and likely non-functional variants of the same proteins (133 and 81 amino acids). Other toxins, including toxic shock syndrome toxin-1 (*tsst-1*) and exfoliative toxin A (*eta*) were rare, only detected in a single and two strains of ST2250 accordingly.

The enterotoxin profiles of MRSArg strains also varied between strains and STs. The most common types included *set17*, *set20*, *set22*, *set25* and *set26* ( ≥ 89.8%), followed by *set23* (25.5%) and *set24* (10.2%), while *seb*, *sec*, *selk*, *sell* and *selq* were infrequent ( ≤ 2.5% of strains). Notably, *set23* was only detected in ST1223 and ST2793 (*p* < 0.01), *set24* was exclusively found in ST1223 (*p* < 0.01), and *set26* was significantly underrepresented in ST1223 isolates (*p* < 0.01).

## Discussion

### Epidemiology and trends

*S. argenteus* was first identified in 2002 in Northern Australia as *S. aureus* CC75 (Ng Jacklyn et al., 2009), and subsequently recognized as a distinct species in 2015 (Tong et al., 2015). Although retrospective investigations into misclassified *S. argenteus* remain relatively scarce, available data demonstrate a global distribution with pronounced regional variation in prevalence. Several studies have suggested geographic “hotspots” for *S. argenteus*, particularly in Southeast Asia, Northern Australia, and the Amazon basin (Yan et al., 2025). In Japan, prevalence has been reported at 6.4%, with retrospective analyses tracing the earliest detection back to 1986 in food-related samples (Ichikawa et al., 2025). In contrast, European data indicate sporadic occurrence and generally lower prevalence rates (<1%). Reports from Sweden (Tang Hallback et al., 2018), Denmark (Hansen et al., 2017), the Netherlands (Witteveen et al., 2022), Belgium (Argudín et al., 2016) and Germany (Alhussein et al., 2025) describe occasional isolates identified within MRSA and MSSA strain collections, dating from the mid-2000s and later. However, as demonstrated in this study, the prevalence of *S. argenteus* remains vulnerable to misclassification through routine diagnostic techniques such as MALDI-ToF. Even advanced methods including whole-genome sequencing and taxonomic classification may fail to fully resolve species identity, indicating that current estimates of prevalence are likely conservative and may underestimate the true occurrence of *S. argenteus* in clinical collections.

During the study period (2008–2019), MRSArg in Norway was predominantly associated with carriage in the community setting. Notably, the percentage of infections was significantly lower than for MRSA (24 and 36% accordingly, *p* < 0.001), and no invasive infections were reported. These findings may thus suggest that *S. argenteus* has a lower pathogenic potential than MRSA. However, other factors such as the limited size of the strain collection and the fact that it is primarily young and healthy individuals that are the main source of import into Norway, makes it difficult to make any broad statements regarding pathogenicity.

Despite uncertainty surrounding the inferred site of acquisition, MRSArg displayed significant associations to international acquisition—especially from the Philippines. This is a country with a relatively high number of immigrants to Norway in the study period.^1^ Although the Philippines has not been frequently reported as a source previously, it has a high prevalence of MRSA (Masim et al., 2021), which may include misclassified MRSArg. It is also located in Southeast Asia—one of the three geographical hotspots for *S. argenteus* described by Becker et al. (2019).

A notable proportion of cases identified in our study involved individuals employed in healthcare settings, representing a novel observation not previously documented in the literature. This is likely also the reason for a relatively high proportion of female cases of MRSArg, as women constitute more than 80% of the workforce in the health and social care sector in Norway (Statistisk Sentralbyrå, 2024). However, our broad definition of healthcare-associated MRSArg, necessitated by missing admission-time data, may have misclassified some cases. International criteria distinguish hospital-onset, healthcare-associated community-onset, and community-associated infections by timing ( > 48–72 h) and prior exposure, meaning admission diagnoses often reflect community acquisition (Pantosi and Venditti, 2009). Thus, our approach likely overestimated HA-MRSArg and underestimated CA-MRSArg. We acknowledge this limitation and recommend future studies incorporate admission-time data or sensitivity analyses to align with standard definitions (Pantosi and Venditti, 2009). Notably, the prevalence of MRSArg among HCWs in this cohort significantly exceeded that reported for MRSA within a comparable population, as previously described (Ronning et al., 2024), using the same broad definition of HA. These findings could thus be indicative of MRSArg presenting an increased risk for nosocomial transmission and outbreaks. However, in this study only two strains were registered as outbreak-related, and furthermore, no evidence of cryptic transmission was uncovered based on cgMLST and cgSNP analyses.

The first confirmed case of MRSArg in Norway was from 2008. Nonetheless, earlier cases may have gone undetected, as national MRSA surveillance incorporating *spa*-typing of isolates was only established that same year. The identification of the initial MRSArg isolate in Norway coincides with its first reported detection in the Netherlands in 2008 (Witteveen et al., 2022), and occurred four years after the earliest known isolate belonging to the “*Staphylococcus aureus* clonal complex 75” was collected in Australia (McDonald et al., 2006). Temporal trends showed a consistent increase in MRSArg detections over the study period, culminating in a peak of 35 cases in 2019. Correspondingly, the incidence increased from 0.04 cases per 100,000 population in 2008 to 0.7 in 2019. The upward trend may reflect multiple introductions over time, rather than ongoing endemic circulation domestically. This is a substantially lower incidence compared to that of MRSA (from 14.2 in 2008 to 48.6 in 2017) in Norway in a comparable time period (Ronning et al., 2024).

Genotyping revealed limited genomic diversity of MRSArg in Norway, with only three main clusters corresponding to ST2250, ST2793 and ST1223, with t6675/ST2250 as the dominant lineage (50.6%). This is in line with previous studies describing the population structure of *S. argenteus* (Goswami et al., 2021). We observed an expansion in *spa*-type diversity after 2014, which may reflect introduction of novel lineages, expansion of community spread or evolutionary adaptation within the local bacterial population.

### Enrichment, screening and rapid detection

In this study, we tested two enrichment broths and four chromogenic MRSA media that all supported growth of MRSArg. Endpoint growth assessment was chosen as it provides a robust, reproducible, and clinically relevant measure of medium performance by reflecting maximum biomass yield for reliable detection across diverse MRSArg genotypes. However, endpoint growth assessment has limitations, as it does not capture growth kinetics and may obscure subtle differences in strain performance or medium characteristics. Of the broths tested, PHMB yielded the highest stationary-phase cell concentrations (2.67 × 10^8^ CFU/mL), outperforming both TSB and TSBM, proving to be a highly effective medium for enhancing MRSArg recovery. Of the chromogenic media, only chromID MRSA SMART agar allowed for practical visual differentiation of MRSArg colonies from MRSA colonies based on a distinct blue coloration of MRSArg colonies after 48 h. Moreover, chromID MRSA SMART agar yielded the highest growth across all tested genotypes, indicating superior sensitivity and positioning it as the most effective medium for MRSArg cultivation among those evaluated.

All tested strains across diverse sequence types yielded positive results using the Xpert^®^ SA Nasal Complete assay, thus allowing for rapid molecular detection of MRSArg, albeit without differentiation from MRSA. While this cross-reactivity poses a risk for misclassification in clinical settings without further species-level identification, is also ensures broad detection of methicillin-resistance within the *S. aureus* complex, in line with recommended reporting practices from the ESGS position paper (Becker et al., 2019): Routine reporting should not differentiate between *S. aureus*, *S. argenteus*, and *S. schweitzeri* unless clear evidence emerges of divergent pathogenicity or clinical outcomes. Importantly, methicillin-resistant isolates, regardless of species within the *S. aureus* complex, should be managed according to MRSA protocols to ensure patient safety and appropriate clinical communication. Another important aspect is that this tool can be utilized for the rapid detection or exclusion of MRSArg during outbreak investigations, thereby supporting timely diagnostic clarification and infection control measures.

### Identification and genotyping

A notable finding in this study is that reliable identification of *S. argenteus* still remains a challenge, both with standard diagnostic methods such as MALDI-TOF MS and with bioinformatic methods like Kraken2, leading to difficulties in distinguishing *Staphylococcus argenteus* from closely related species such as *S. aureus* and *S. schweitzeri*. These issues likely stem from low representation of *S. argenteus* genotypes as well as other *S. aureus* complex species in the underlying databases (Chen et al., 2018; Eshaghi et al., 2021). A more general issue may be persistent misclassification in databases. This diagnostic ambiguity prompted the development of molecular tools for accurate species-level detection and genotyping within this study.

A species-specific multiplex PCR assay targeting the *S. argenteus*-specific gene *yhfT*, the methicillin resistance gene *mecA*, and the virulence-associated gene *lukE* was developed and validated. This assay enabled simultaneous detection of species identity, beta-lactam resistance, and virulence marker, offering a rapid and comprehensive molecular diagnostic tool. In concordance with the WGS data, MRSArg was fully distinguished from MRSA. Such assays are critical for overcoming the limitations of conventional phenotypic methods and ensuring accurate identification of MRSArg in both clinical and surveillance contexts. To further enhance diagnostic precision, development and implementation of novel primers tailored to *S. argenteus* enabled successful Sanger sequencing-based *spa*-typing across all isolates, achieving 100% type ability. This represents a significant methodological advancement, facilitating robust strain-level resolution and epidemiological tracking of MRSArg.

### Genomic diversity, antibiotic resistance and virulence factors

Whole genome sequencing highlighted the limited genomic diversity of MRSArg strains across the study collection, with only three distinct clades corresponding to ST2250, ST2793, and ST1223. Core genome SNP analysis furthermore revealed low degree of genetic diversity within the major MRSArg STs identified. Although the dataset comprised strains collected from diverse geographic regions and over an extended time period, the overall genetic diversity was thus relatively low compared to, for instance, *S. aureus* (Liu et al., 2023), likely reflecting the much more recent and limited spread of MRSArg. Following the evolutionary trajectory and potential clonal success of MRSArg may thus provide valuable insights into the emergence and adaptation of a *S. aureus*-like pathogen.

The antimicrobial resistance profiles of the MRSArg isolates analyzed in this study revealed that antimicrobial resistance to other classes than beta-lactams was notably low, which is in line with previous studies (Witteveen et al., 2022). Consequently, standard treatment regimens commonly applied for MRSA are expected to remain effective, with low risk of treatment failure for MRSArg infections. No adapted or pathogen-specific therapeutic strategies appear necessary for MRSArg based on the phenotypic antibiotic susceptibility testing and antibiotic resistance genes detected in this study. MRSArg strains however, shared many notable virulence factors with *S. aureus*, many of which are associated with mobile genetic elements like plasmids, phages, pathogenicity islands (Wu et al., 2021), indicating potential horizontal spread.

While the MRSArg strains shared many virulence determinants, we did observe some ST-specific variability, especially in toxin genes. While many MRSA encode PVL, a cytotoxin associated with increased virulence of *S. aureus*, this was not detected in any MRSArg strain in this study. Instead, *S. argenteus* possessed analogous genes encoding a distinct leukocidin most closely related to LukED; however, its role in infection remains less well-described. Notably, these genes were misclassified by some tools/databases as *lukS/lukF-PVL*, which may lead to erroneous conclusions about the prevalence and role of PVL in *S. argenteus*.

## Conclusion

This study provides the first comprehensive overview of methicillin-resistant *Staphylococcus argenteus* in Norway, covering an extended study period. Our results reveal a predominantly community-associated carriage pattern with strong links to international acquisition, particularly from Southeast Asia. MRSArg appears to have a lower infection potential than MRSA within the Norwegian context, though limited data and import-specific patterns warrant cautious interpretation. The unexpectedly high prevalence of HA-MRSArg, particularly among healthcare workers, however, underscores the potential for nosocomial transmission and outbreak risk. Diagnostic challenges—stemming from phenotypic similarity to *S. aureus* and limitations of conventional tools and databases—were addressed through the development of a species-specific multiplex PCR targeting *yhfT*, *mecA*, and *lukE* and adapted *spa*-typing primers, enabling specific molecular identification and genotyping of MRSArg.

## Materials and methods

### Study population

The background population included all strains of MRSA and MRSArg from the Norwegian MRSA reference laboratory strain collection covering the years 2008–2019 (*n* = 18,152). Inclusion criteria for the *S. argenteus* study population included all strains with inconclusive *spa-*typing (“non-typable MRSA”), multilocus sequence types (STs), characteristic repeats, alleles, and clonal complexes that have previously been associated with the species. Strains meeting either of these genetic criteria were presumptively identified as *S. argenteus* and selected for further investigation.

### Clinical and epidemiological data

Epidemiological data on all included cases was collected from the Norwegian Surveillance System for Communicable Diseases (MSIS) and the request forms from the referring laboratory or the treating physician (accessed through the laboratory information system). The Information from MSIS included age, sex, county of residence, country of birth, parent’s country of birth, admission to hospital or nursing home, reason for sampling, place of acquisition, part of a known outbreak (MRSA request forms or Norwegian outbreak rapid alert system Vesuv) and if the person worked within the health care system. The data obtained from the laboratory information system included sample date, sampling site and results of laboratory analyses. All MRSArg cases were categorized as carriage, infection, invasive infection or unknown based on sampling site/type of sample. Age groups were based on categories defined by Diaz et al. (2021). Due to lack of data on admission time for hospitalized patients, we used a broad definition of healthcare-associated MRSArg (HA-MRSArg). A case was classified as HA-MRSArg if diagnosed during admission to a hospital or nursing home and/or if MRSArg was diagnosed in healthcare workers (HCWs), while community-acquired MRSArg (CA-MRSArg) was defined as all other cases.

### Bacterial strains and growth assays

Bacterial strains were routinely cultured on blood agar at 35°C. Four commercially available chromogenic media designed for the selective identification of MRSA were evaluated using a representative panel of MRSArg strains (*n* = 25), encompassing all MRSArg *spa*-types (one strain per *spa*-type) included in the study (Table 5). The media included chromID MRSA SMART (BioMérieux), Scientific Brilliance Scientific Brilliance MRSA 2 (Thermo Fisher Scientific), BD BBL CHROMagar MRSAII (BD) and CHROMagar MRSA (CHROMagar). Plates were inspected after 24- and 48-h incubation and growth qualitatively assessed as sparse, moderate or abundant.

Two enrichment broths were additionally evaluated in this study; tryptic soy broth (TSB) supplemented with aztreonam (20 mg/L) and cefoxitin (3.5 mg/L) (TSBM), and phenol red mannitol broth supplemented with ceftizoxime (5 mg/L) and aztreonam (75 mg/L) (PHMB). Briefly, MRSArg strains were cultured at 35°C for 24 h in unsupplemented TSB and then diluted (100 μL) in 5 mL enrichment broth. After 24 h incubation at 35°C, serial dilutions were plated on blood agar plates, before semi-quantitative growth assessment after 24- and 48-h incubation.

### Antimicrobial susceptibility testing

Antimicrobial susceptibility testing (AST) was performed for all included MRSArg stains (*n* = 160) at the Norwegian MRSA reference laboratory (2009–2015) or the referring laboratory (2016–2019) using the European Committee on Antimicrobial Susceptibility Testing (EUCAST) disc diffusion method or agar gradient method as previously described (Ronning et al., 2025). Results were interpreted as either susceptible, intermediate or resistant using the clinical breakpoints for *S. aureus* according to the 2017 EUCAST break point table v.7.1.

### MALDI-TOF MS identification of methicillin-resistant *S. argenteus*

Identification of a selection of bacterial strains (*n* = 25) was performed using matrix-assisted laser-desorption/ionization time-of-flight mass spectrometry (MALDI-ToF MS) (Clark et al., 2013), with a MALDI Biotyper Sirius instrument and the Bruker Daltonics database V12.0.0.0_10833-11897, on single colony obtained from sheep blood agar COLS+ (Oxoid). Log (score) values above 2.0 were accepted as probable species-level identification, while scores between 1.7 and 1.9 were accepted as probable genus-level identification (Sauer et al., 2008).

### Rapid detection of methicillin-resistant *S. argenteus*

Rapid detection of MRSArg was performed using the Xpert SA Nasal Complete on a GeneXpert XVI instrument according to the manufacturer’s recommendations. Due to costs and reagent availability, a selection of MRSArg strains (*n* = 7) from different *spa*-types, representing all three STs detected in this study, were used for GeneXpert testing.

### Whole genome sequencing

All 160 MRSArg isolates were subjected to whole genome sequencing. Briefly, colonies were suspended in TE-buffer and treated with proteinase K (2 mg/mL) and lysostaphin (0.1 mg/mL) for 15 min with shaking at 37°C, before heating for 15 min at 65°C. Genomic DNA was then isolated using the EZ1 DNA tissue kit with an EZ1 Advanced XL instrument (Qiagen). Sequencing libraries were prepared using the Nextera XT sample prep kit and sequenced on the MiSeq platform with MiSeq v3 reagents, with 300 bp paired end reads (Illumina). Raw data were quality controlled, trimmed/filtered, classified with Kraken2 (PlusPF database) and *de novo* assembled, and the assembled genomes annotated, and typed (multi-locus sequence typing, MLST, according to *S. aureus* pubMLST scheme) with the Nullarbor pipeline version 2.0 (Seemann et al., 2018). Three strains were excluded from further bioinformatic analyses based on quality control. Assemblies are available from GenBank under BioProject PRJNA1306038.

Resistance genes were identified using AMRFinder+ with the NCBI AMRFinder+ database (Feldgarden et al., 2021). Virulence genes were identified using ABRicate (Seemann, 2016) with the Virulence Factor DataBase (VFDB) (Chen et al., 2005). Additionally, SCCmecFinder 1.2 was used for SCC*mec* typing of all strains (Kaya et al., 2018). The core and accessory genome of the strains was defined and a core genome alignment produced by Roary version 3.13 (Page et al., 2015). Fasttree 2.1.10 (Price et al., 2010) was used to infer a maximum likelihood core genome phylogeny with the GTR model. Distance estimation was performed by Molecular Evolutionary Genetics Analysis (MEGA) software (Tamura et al., 2021), with genetic distances referring to the number of SNPs in the core genome alignment. SeqSphere+ (Ridom) was used for creating a minimum spanning tree (MST) of *spa*-repeats. Visualizations of phylogenies with metadata were created using iTOL (Letunic and Bork, 2021).

### Development of a multiplex PCR for detection of methicillin-resistant *S. argenteus*

A multiplex real-time PCR for the specific detection of methicillin-resistant *S. argenteus* was developed in this study (Table 5).

Species-specific candidate genes for PCR assay development were identified using Roary (Page et al., 2015) by performing pan-genome analysis. The dataset comprised reference genomes including *S. argenteus* (*n* = 16), *S. aureus* (*n* = 21), and other stapylococcal species (*n* = 53) including *Staphylococcus schweitzeri* strain NCTC13712, as well as the complete *S. argenteus* strain collection from this study (Supplementary Table 5). A clustering threshold of 70% identity was used to avoid subclustering of orthologous proteins from different staphyloccoccal species. Potential targets were then selected based on presence in all *S. argenteus* genomes, absence in all other staphylococcal genomes, presence in single copy and single fragment per genome, and within cluster identity of ≥ 97%. Additionally, for each candidate gene, BLASTn against the non-redundant NCBI database should only yield hits against *S. argenteus*. From this, a putative acyl–CoA ligase encoding gene, *yhfT*, was selected as target.

Based on virulence profiles of this MRSArg strain collection, the bicomponent leukotoxin LukED was selected as the secondary target for the species-specific PCR. The sequences of the *lukD* and *lukE* genes were extracted from *S. argenteus* reference genomes (*n* = 16) and the MRSArg strain collection (*n* = 160). A multiple alignment was then used to identify conserved regions suitable for primers and probes, after which *lukE* was selected as the secondary target for the multiplex PCR. Primers and probes for *yhfT* and *lukE* were then designed using IDT RealTime qPCR Assay (Table 5).

### Multiplex PCR validation

The PCR primers and probes for *yhfT* and *lukE* were tested, in triplex with *mecA*, against a selection of whole genome sequenced MRSArg (*n* = 20) and MRSA strains (*n* = 20) of different genotypes for validation. Validation was performed by assessing diagnostic performance, including concordance WGS, lack of cross-reactivity and comparison of two DNA extraction methods; boiled lysates and EZ1 DNA extraction. The sensitivity (limit of detection) and amplification efficiency of the multiplex PCR was furthermore assayed, using *S. argenteus* t6675/ST2250 strain SO-SARG8-1.

For validation, two DNA preparation methods were compared. Ten MRSArg and ten MRSA isolates were processed as boiled lysates, prepared by suspending colonies in molecular-grade water, heating at 95°C for 15 min with shaking (300 rpm), centrifuging (14,500 rpm for 2 min), and collecting the supernatant. For another ten MRSArg and ten MRSA isolates, DNA was extracted with the EZ1 Advanced XL instrument from WGS. All samples were tested in duplicate by real-time PCR on the QuantStudio 5 system, using Perfecta Custom Multiplex qPCR SuperMix, an annealing temperature of 62°C, and 35 cycles.

#### Genotyping (*spa*-typing) of *S. argenteus*

Primers commonly used for *spa*-typing of *S. aureus* have several mismatches relative to the *S. argenteus spa*-region, often causing sequencing failure or low-quality results. Sequences of the *S. argenteus spa*-region were therefore extracted from *S. argenteus* reference genomes (*n* = 16) as well as the MRSArg strain collection (*n* = 160). A multiple alignment with MUSCLE (Alhussein et al., 2025) was then used for designing primers specifically targeting the binding sites within the *spa*-gene of *S. argenteus*: spa-1113arg-f and spa-1514arg-r (Table 5). All strains were subjected to *spa*-typing following the methodology established by Harmsen et al. (2003) using the Ridom StaphType software and Spa Server (Grundmann et al., 2010).

### Statistical analyses

Statistical analyses of discrete variables were performed using Fisher’s exact test. The Benjamini-Hochberg method was used to correct for multiple hypothesis testing, with adjusted *p* < 0.05 regarded as statistically significant.

## Data Availability

The datasets presented in this study can be found in online repositories. The names of the repository/repositories and accession number(s) can be found at: https://www.ncbi.nlm.nih.gov/, PRJNA1306038.

## Footnotes

1
https://www.ssb.no/statbank/table/13880/
