# Supplementary material for: Unmasking Methicillin-resistant Staphylococcus argenteus: pathogenic potential, diagnostic pitfalls and antibiotic resistance of MRSArg in Norway 2008–2019
Source: Front Microbiol. 2026 Jan 14;16:1734191. doi: 10.3389/fmicb.2025.1734191 (PMC12847390; doi:10.3389/fmicb.2025.1734191)
Supplement: Supplementary file 2 [file Image_1.pdf]

Strain	Incubation	Columbia sheep blood, COLS+	chromID MRSA Smart	Scientific Brilliance MRSA2	BD/BBL Chromagar MRSAII	CHROMagar TM MRSA
SO-SARG14-10, t5078 – ST2250	24 h	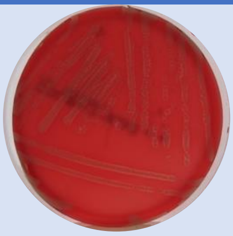	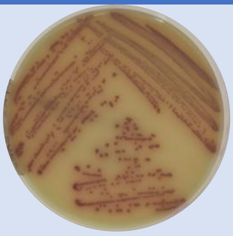	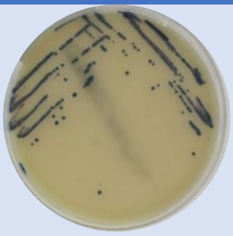	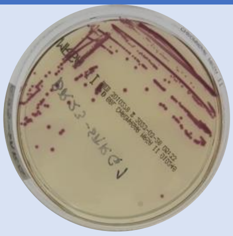	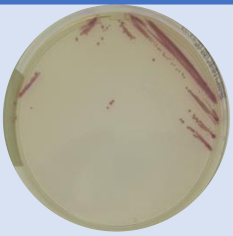
	48 h	-	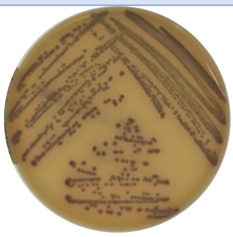	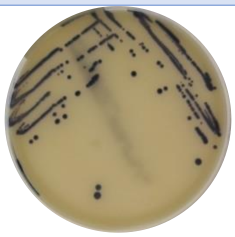	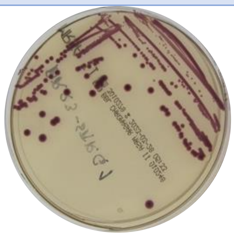	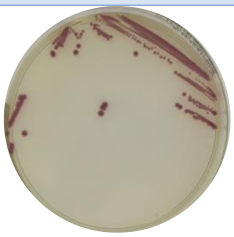
SO-SARG15-10, t6188 – ST2793	24 h	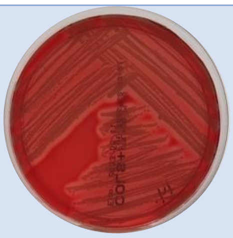	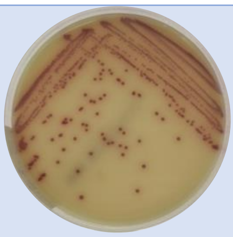	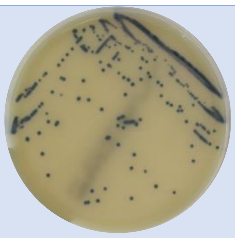	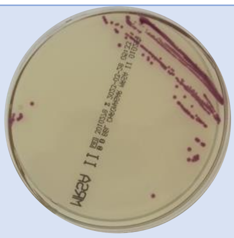	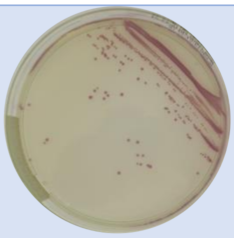
	48 h	-	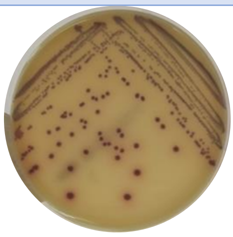	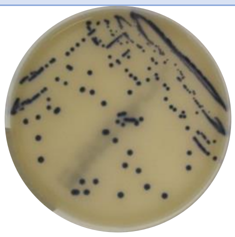	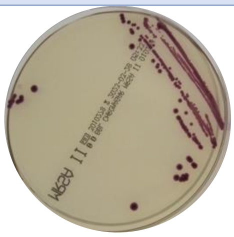	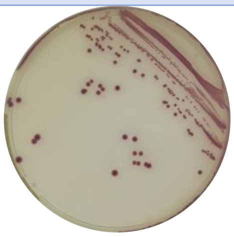
SO-SARG15-1, t6675 – ST2250	24 h	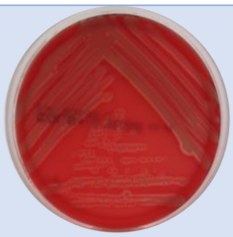	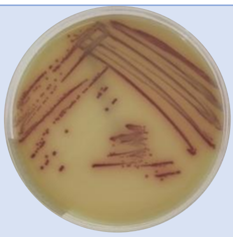	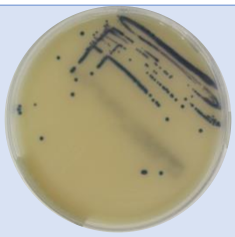	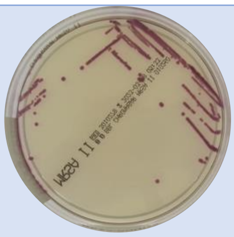	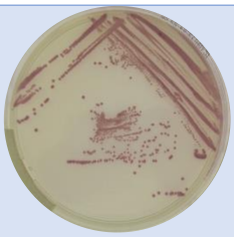
	48 h	-	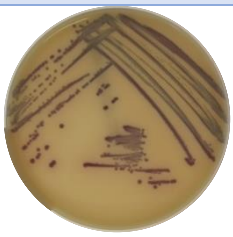	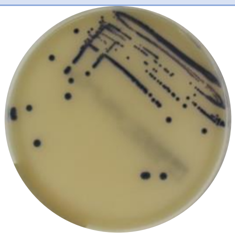	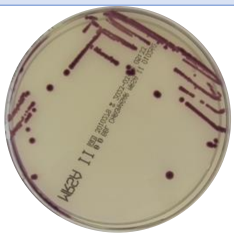	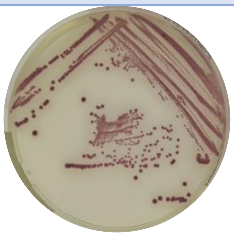
SO-SARG17-9, t6947 –	24 h	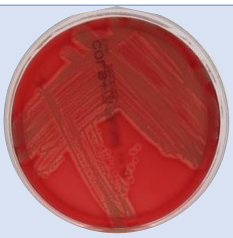	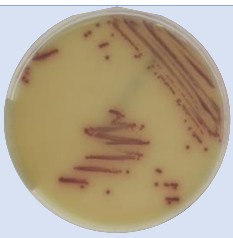	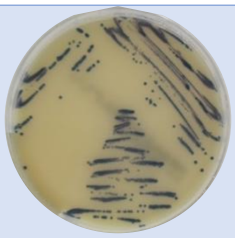	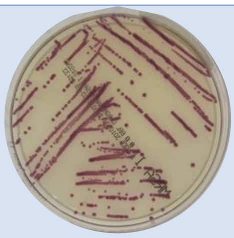	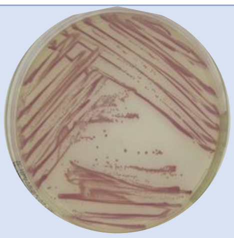

Åpen

**ST2793**	48 h	-	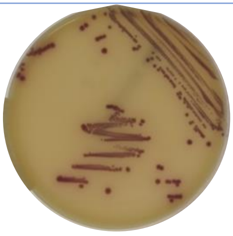	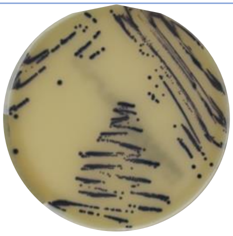	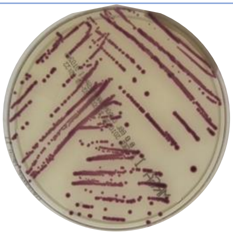	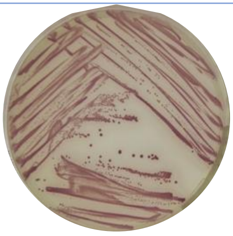
**SO-SARG17-15, t7960 – ST2500**	24 h	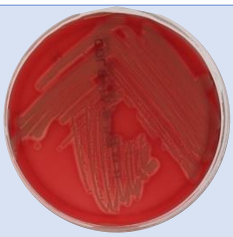	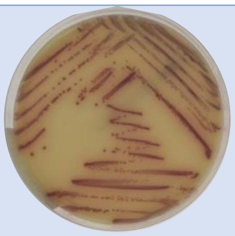	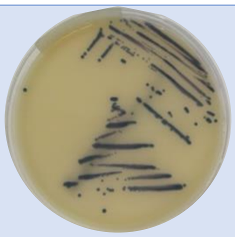	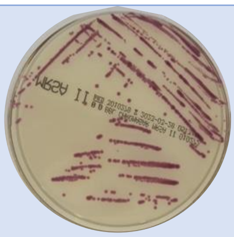	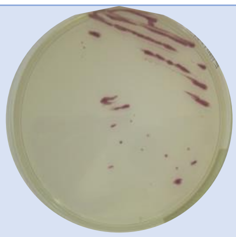
	48 h	-	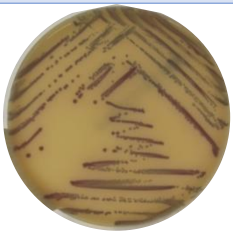	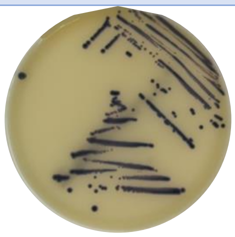	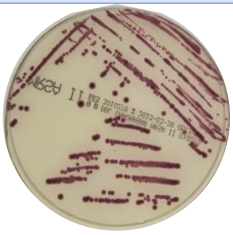	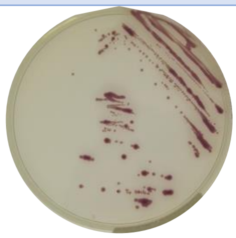
**SO-SARG17-8, t9791 – ST1223**	24 h	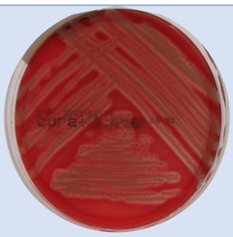	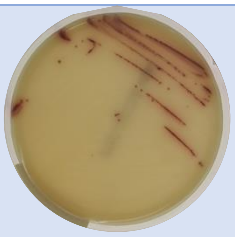	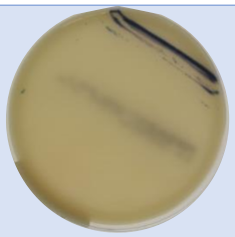	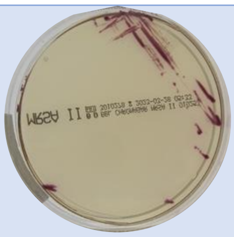	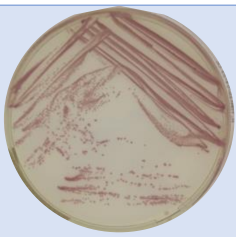
	48 h	-	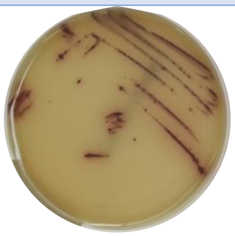	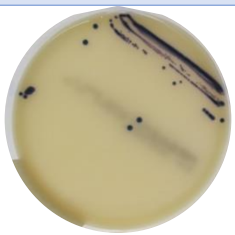	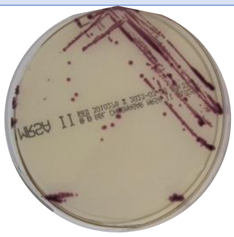	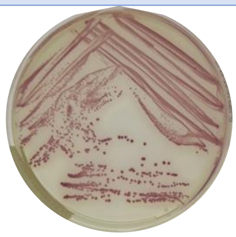
**SO-SARG17-7, t9906 – ST1223**	24 h	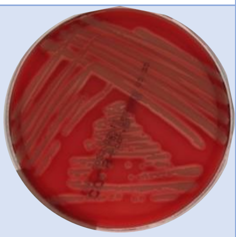	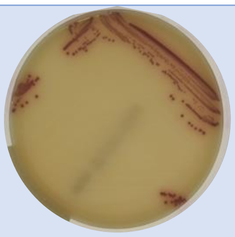	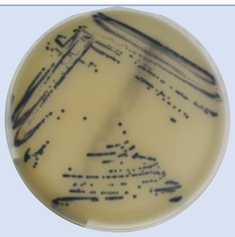	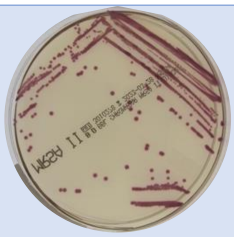	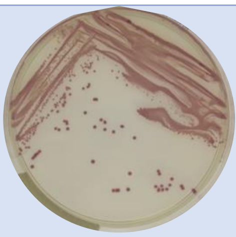
	48 h	-	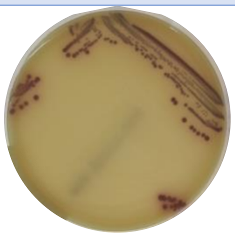	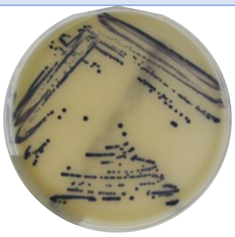	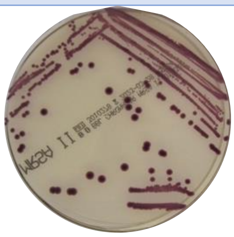	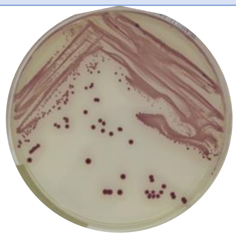

Åpen

**SO-SARG19-4, t10900 – ST2250**	24 h	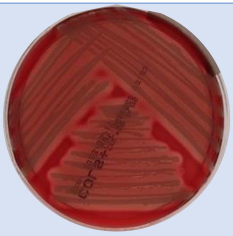	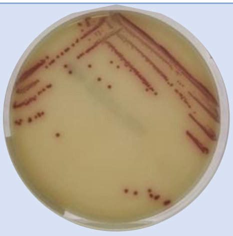	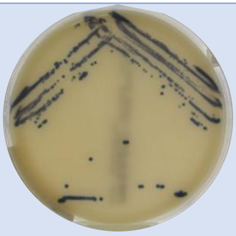	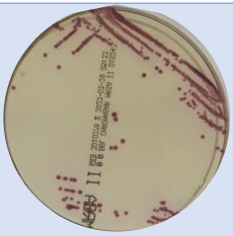	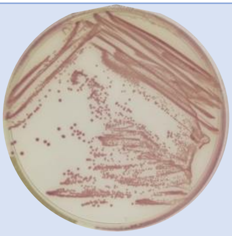
	48 h	-	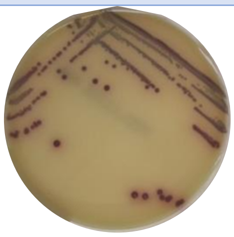	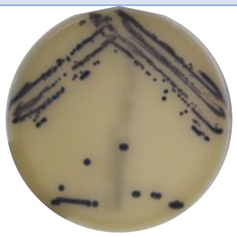	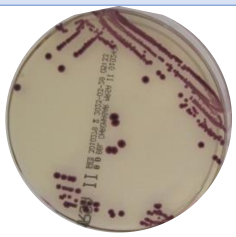	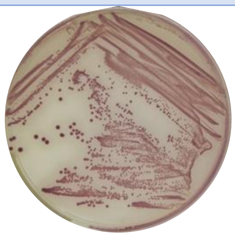
**SO-SARG17-12, t12708 – ST2250**	24 h	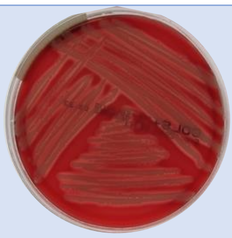	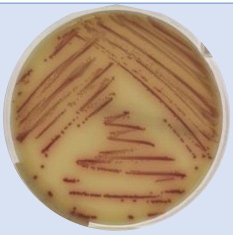	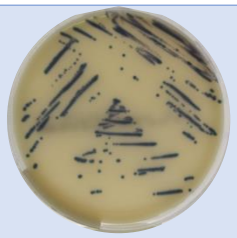	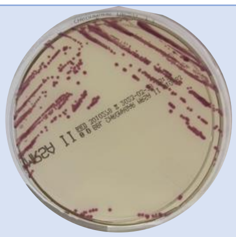	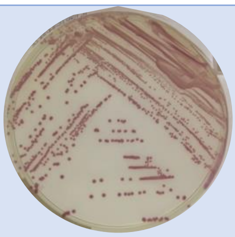
	48 h	-	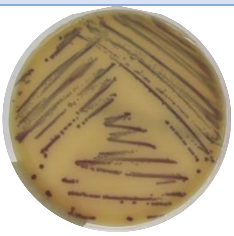	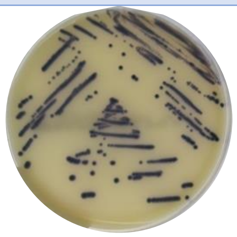	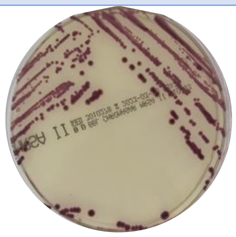	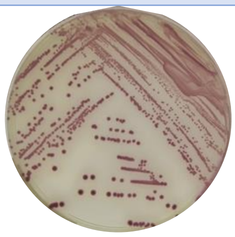
**SO-SARG13-2, t12782 – ST1223**	24 h	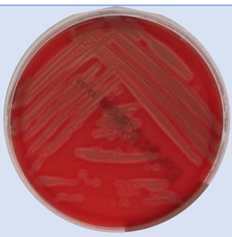	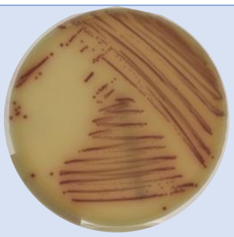	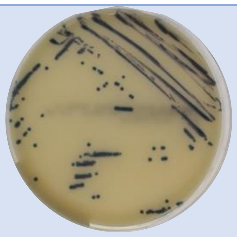	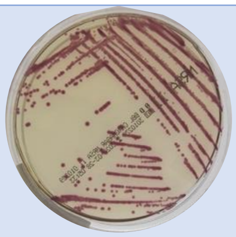	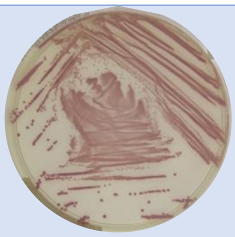
	48 h	-	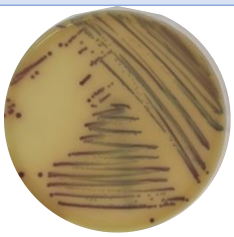	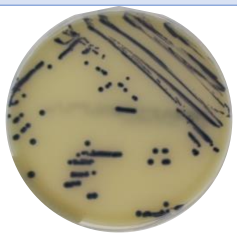	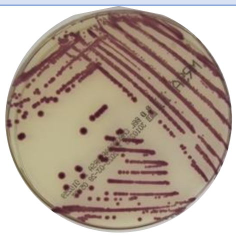	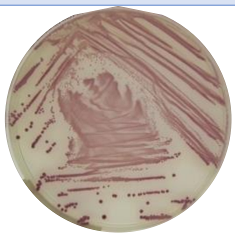
**SO-SARG16-4, t14475 –**	24 h	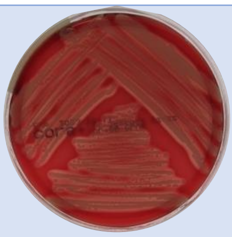	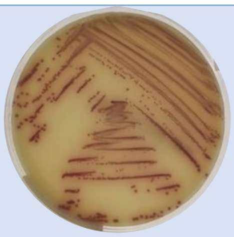	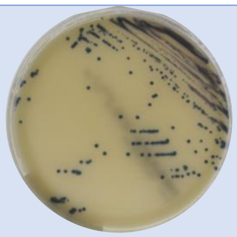	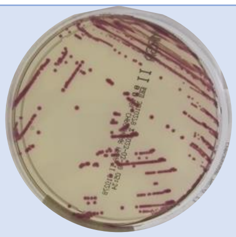	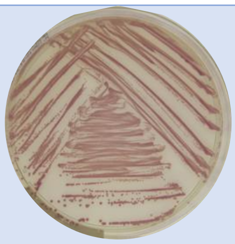

Åpen

**ST2250**	48 h	-	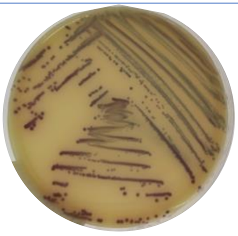	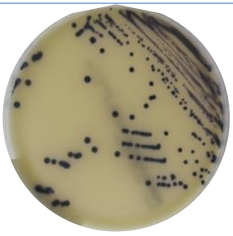	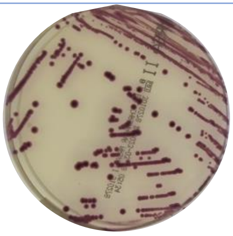	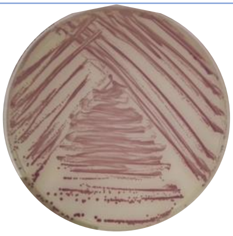
**SO-SARG15-2, t15218 – ST1223**	24 h	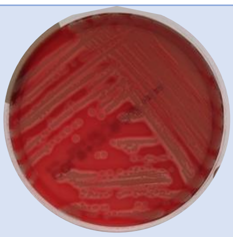	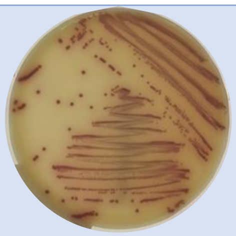	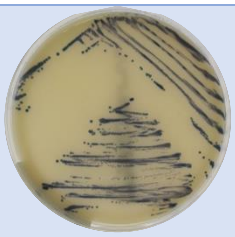	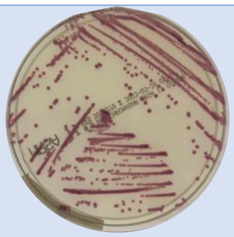	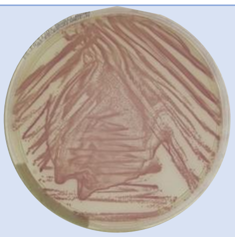
	48 h	-	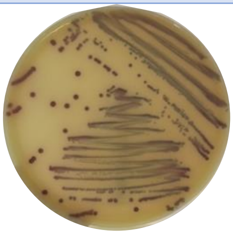	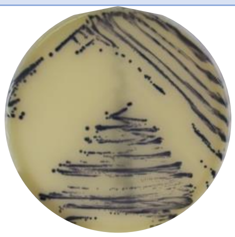	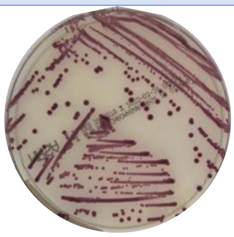	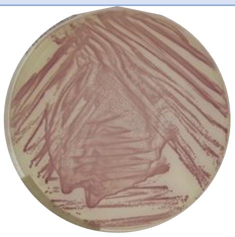
**SO-SARG14-1, t15221 – ST1223**	24 h	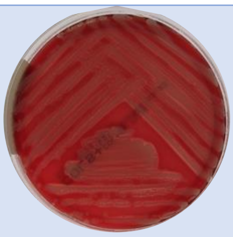	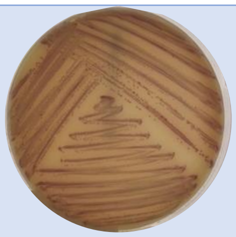	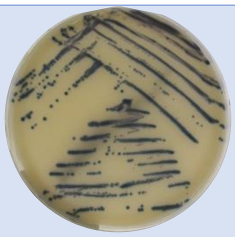	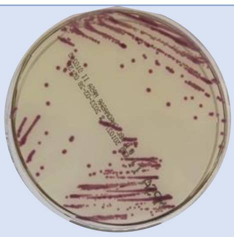	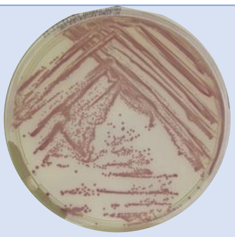
	48 h	-	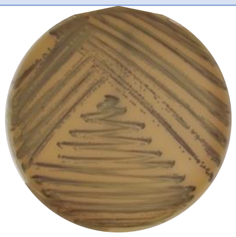	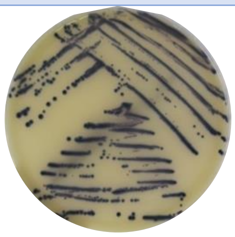	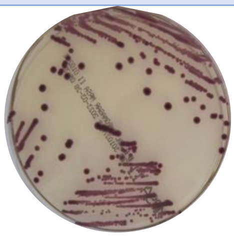	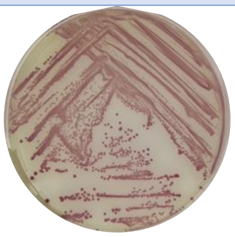
**SO-SARG15-3, t15443 – ST1223**	24 h	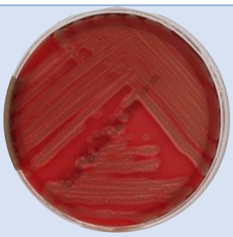	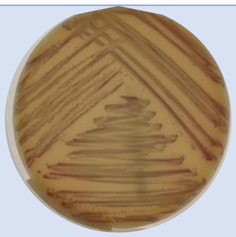	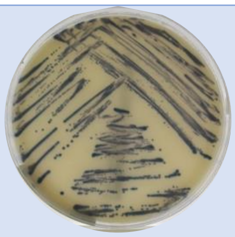	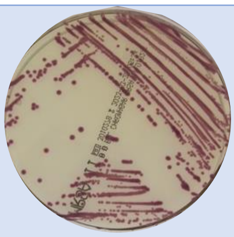	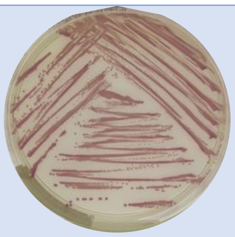
	48 h	-	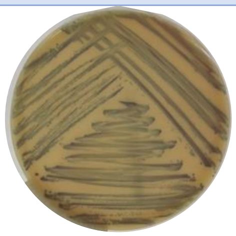	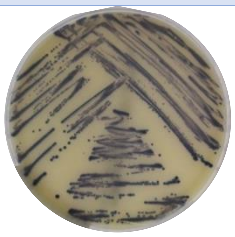	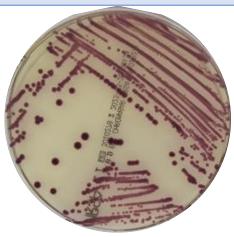	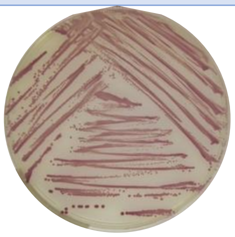

Åpen

**SO-SARG17-4, t16149 – ST2250**	24 h	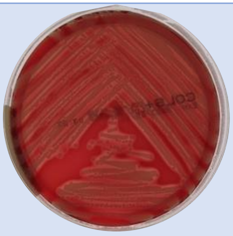	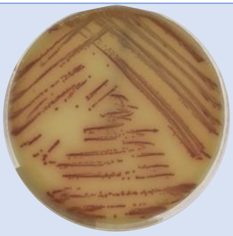	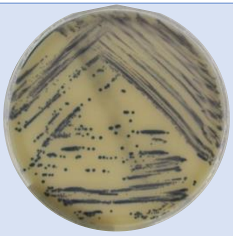	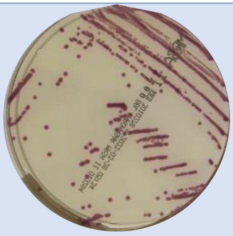	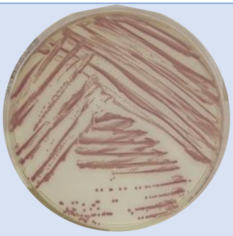
	48 h	-	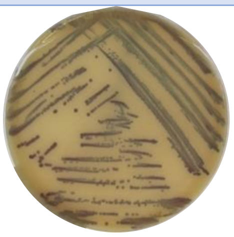	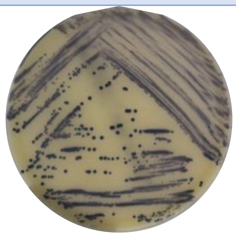	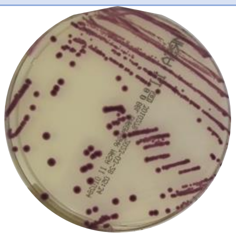	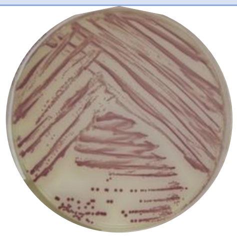
**SO-SARG16-5, t16376 – ST2250**	24 h	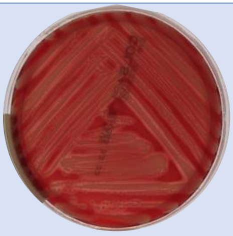	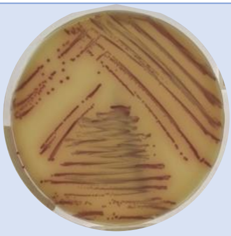	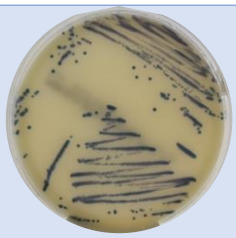	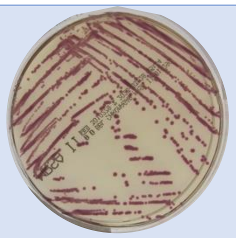	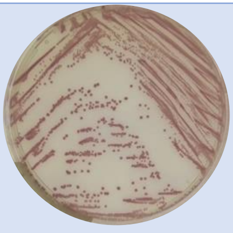
	48 h	-	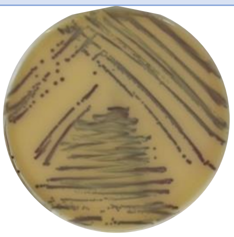	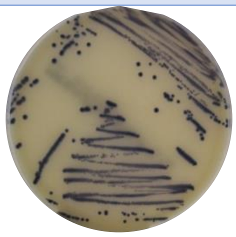	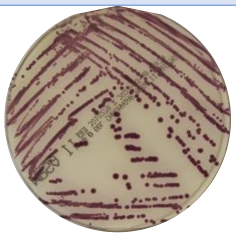	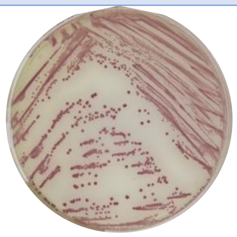
**SO-SARG13-3, t16726 – ST2793**	24 h	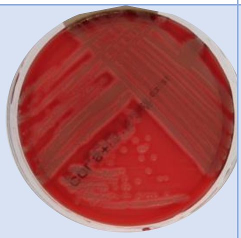	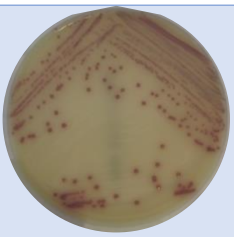	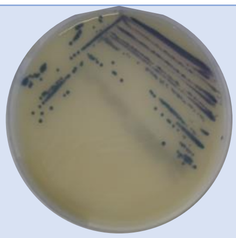	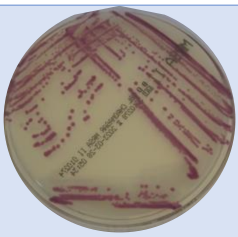	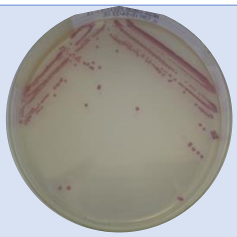
	48 h	-	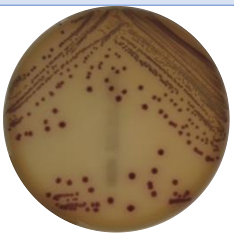	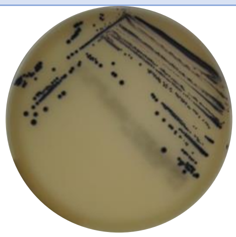	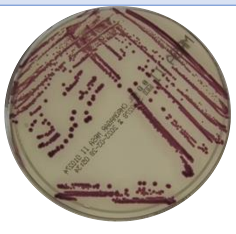	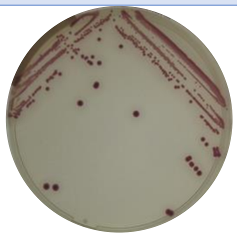
**SO-SARG17-3, t16808 –**	24 h	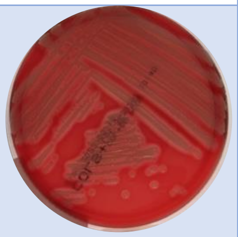	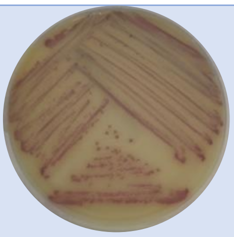	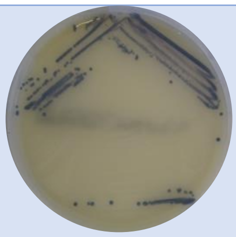	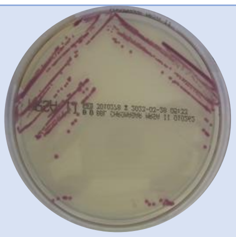	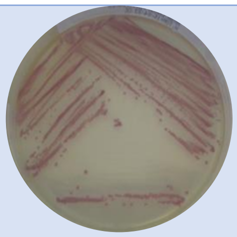

Åpen

**ST2250**	48 h	-	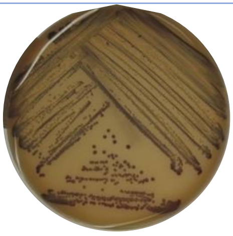	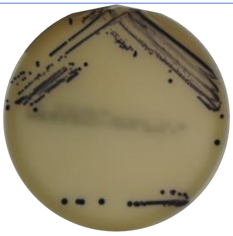	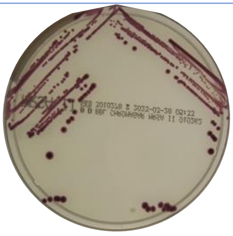	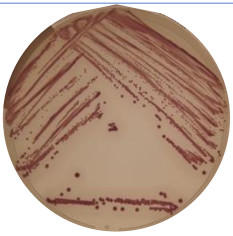
**SO-SARG17-10, t17057-ST2793**	24 h	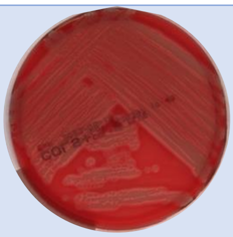	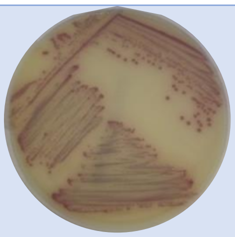	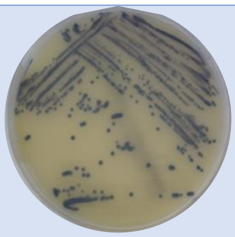	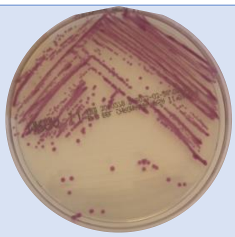	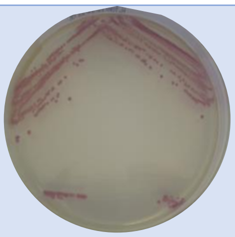
	48 h	-	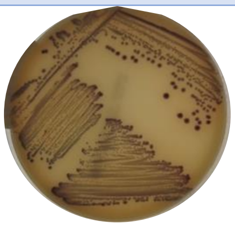	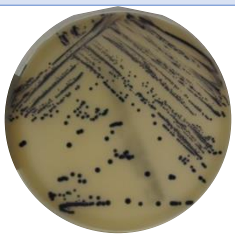	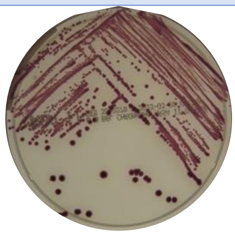	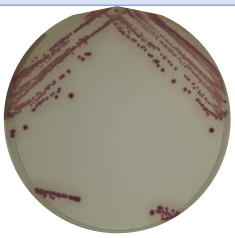
**SO-SARG18-5, t17700 – ST2793**	24 h	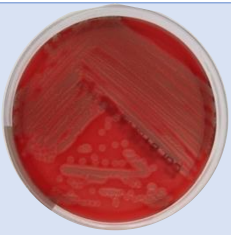	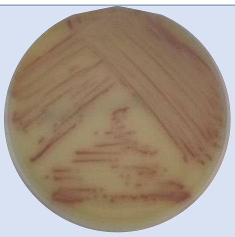	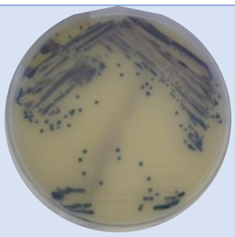	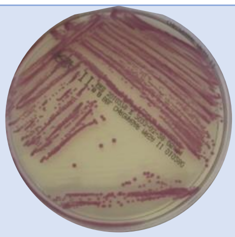	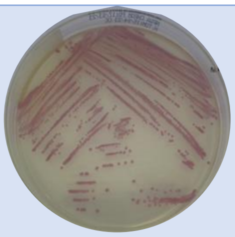
	48 h	-	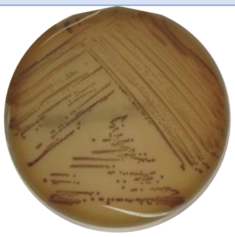	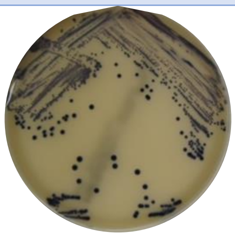	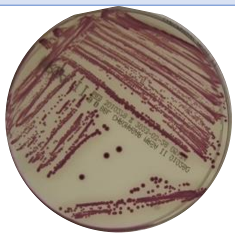	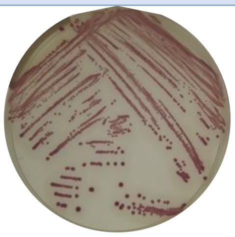
**SO-SARG18-3, t17987 – ST2793**	24 h	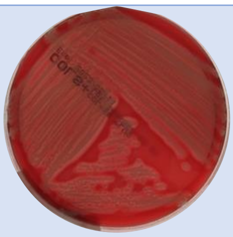	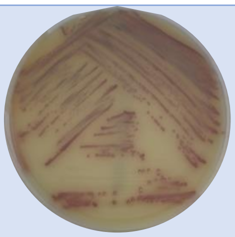	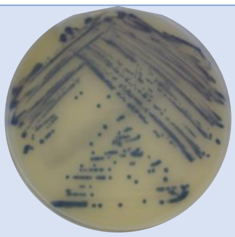	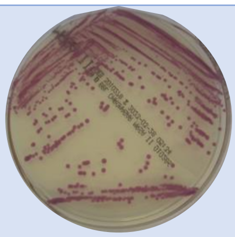	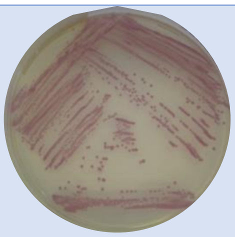
	48 h	-	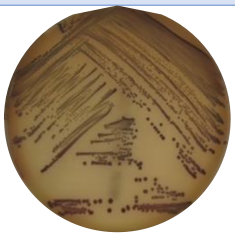	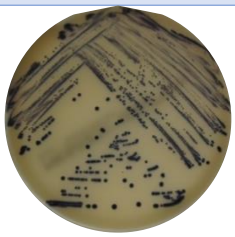	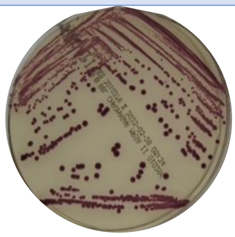	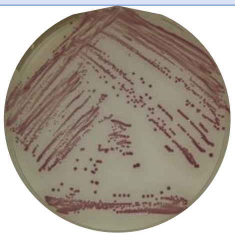

Åpen

SO-SARG19-15, t19056 – ST2793	24 h	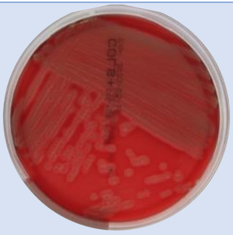	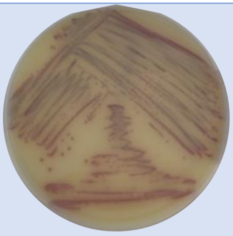	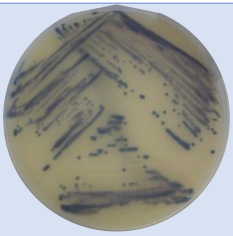	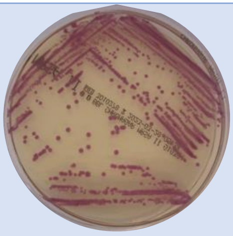	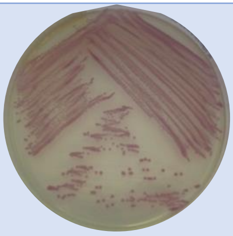
	48 h	-	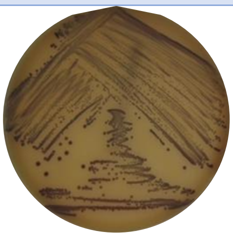	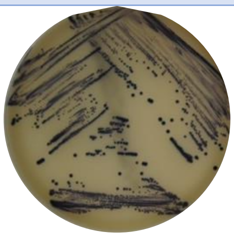	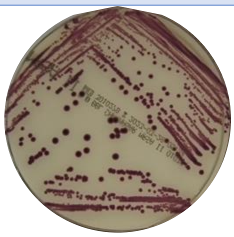	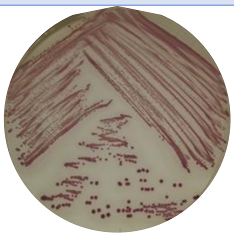
SO-SARG15-4, t20203 – ST2250	24 h	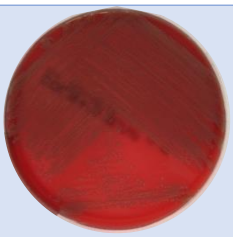	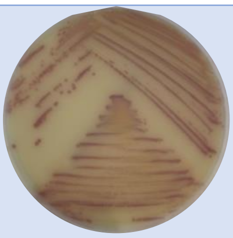	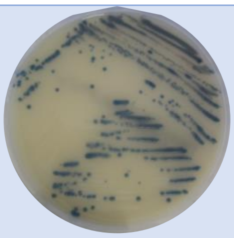	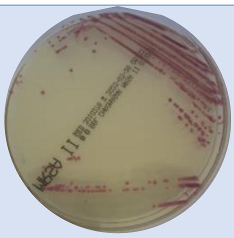	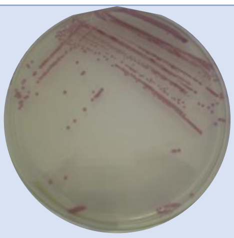
	48 h	-	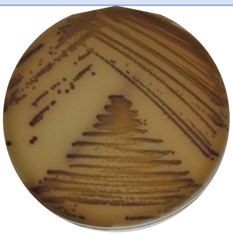	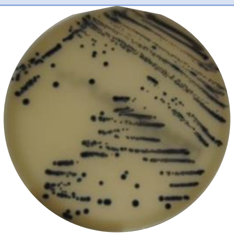	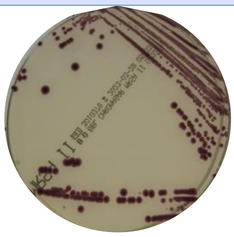	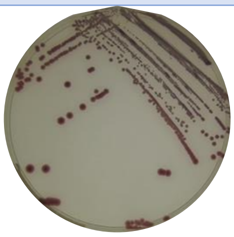
SO-SARG19-18, t20204 – ST2250	24 h	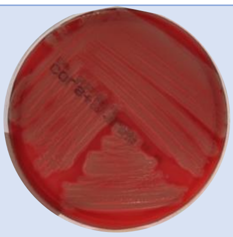	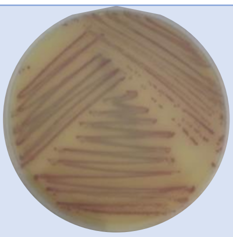	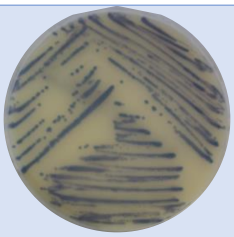	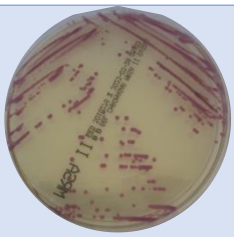	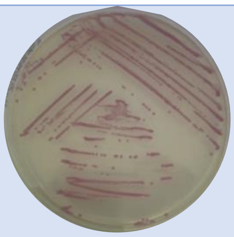
	48 h	-	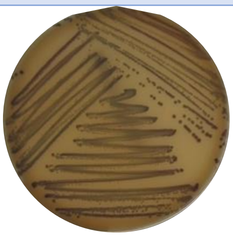	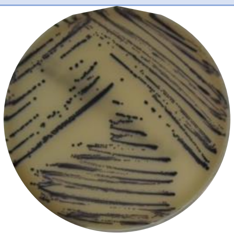	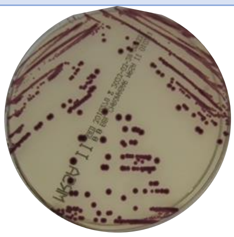	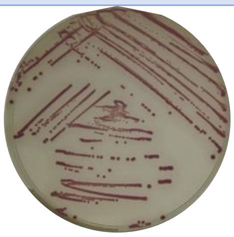
SO-SARG19-11, t20211 –	24 h	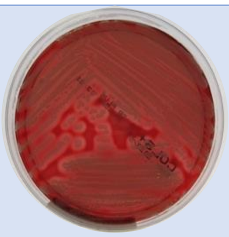	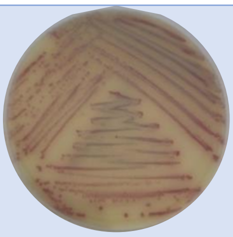	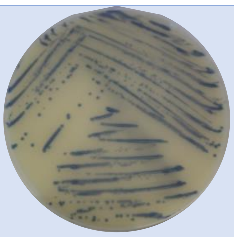	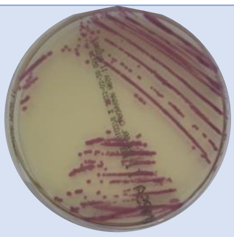	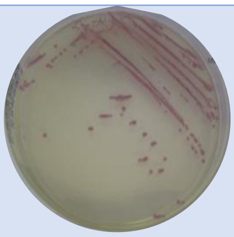

Åpen

**ST2250**	48 h	-	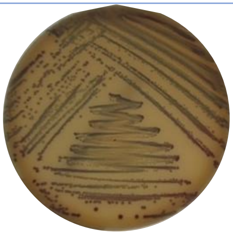	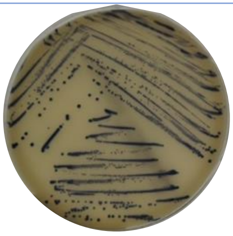	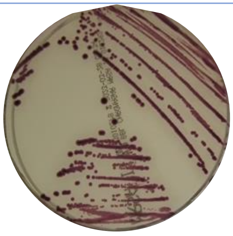	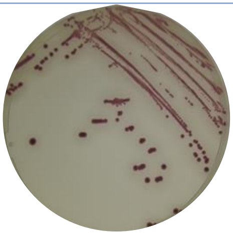

Supplementary figure 1. Growth results for 25 different MRSArg-strains on Columbia sheep blood COLS+, ChromID MRSA Smart, Scientific Brilliance MRSA2, BD/BBL Chromagar MRSAII and CHROMagar TM MRSA. Growth on COLS+ was observed after 24 hours (24 h) incubation, while growth on +, ChromID MRSA Smart, Scientific Brilliance MRSA2, BD/BBL Chromagar MRSAII and CHROMagar TM MRSA was observed after 24h and 48 hours (48 h).

Åpen
